# The dynamic behavior of chromatophores marks the transition from bands to spots in leopard geckos

**DOI:** 10.1073/pnas.2400486121

**Published:** 2024-07-08

**Authors:** Asier Ullate-Agote, Athanasia C. Tzika

**Affiliations:** ^a^Laboratory of Artificial & Natural Evolution, Department of Genetics & Evolution, University of Geneva, Geneva 1211, Switzerland

**Keywords:** skin coloration, leopard gecko, chromatophores, reptiles, PAX7

## Abstract

Reptilian skin coloration is spectacular and diverse, yet little is known about the ontogenetic processes that govern its establishment and the molecular signaling pathways that determine it. Here, we focus on the development of the banded pattern of leopard gecko hatchlings and the transition to black spots in the adult. With our histological analyses, we show that iridophores are present in the white and yellow bands of the hatchling and they gradually perish in the adult skin. Furthermore, we demonstrate that melanophores can autonomously form spots in the absence of the other chromatophores both on the regenerated skin of the tail and on the dorsal skin of the Mack Super Snow (MSS) leopard geckos. This color morph is characterized by uniform black coloration in hatchlings and black spots in adulthood; we establish that their skin is devoid of xanthophores and iridophores at both stages. Our genetic analyses identified a 13-nucleotide deletion in the *PAX7* transcription factor of MSS geckos, affecting its protein coding sequence. With our single-cell transcriptomics analysis of embryonic skin, we confirm that *PAX7* is expressed in iridophores and xanthophores, suggesting that it plays a key role in the differentiation of both chromatophores. Our in situ hybridizations on whole-mount embryos document the dynamics of the skin pattern formation and how it is impacted in the *PAX7* mutants. We hypothesize that the melanophores–iridophores interactions give rise to the banded pattern of the hatchlings and black spot formation is an intrinsic capacity of melanophores in the postembryonic skin.

## Significance

This study focuses on the establishment of the banded pattern of the leopard gecko hatchlings and the transition to spots in the adult. We demonstrate that iridophores are necessary for the formation of bands and that melanophores autonomously form spots in the absence of other chromatophores, both in the regenerating tail and in the Mack Super Snow color morph. We establish that this morph lacks xanthophores and iridophores due to a genetic mutation in the PAX7 transcription factor. With transcriptomic data, we confirm the expression of PAX7 in both xanthophores and iridophores as they differentiate. Our study provides insights into the regulatory mechanisms governing skin pigmentation in leopard geckos and enhances our understanding of color pattern formation in reptiles.

Reptiles display a wide variety of skin coloration which is brought about by the combination of three types of chromatophores in their skin. Melanophores are characterized by their dendritic shape and the presence of melanin in dedicated endosomes, named melanosomes. Xanthophores are responsible for the red/yellow coloration and carry endosomes containing either pteridines or carotenoids. Iridophores hold numerous vesicles filled with guanine crystals, reflecting the light at a specific wavelength when organized and scattering it when disorganized. Depending on the distribution of these chromatophores in the skin, different coloration patterns emerge. As reptiles do not go through metamorphosis, for most species, the coloration pattern is set during embryonic development and remains stable throughout the life of the animal. There are exceptions though with animals continuously changing pattern, notably the green and black scales of the ocellated lizards (1) and other lizards with a scale-by-scale color pattern (2).

**Fig. 1.**
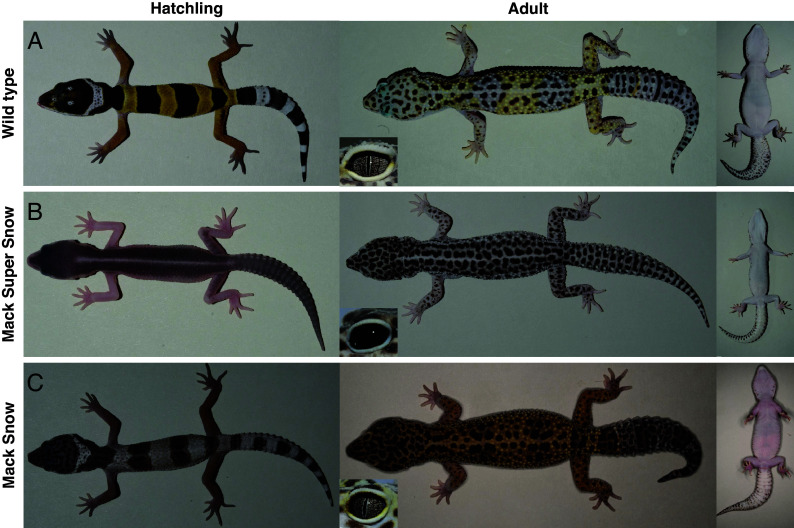
Wild-type and Mack Snow geckos. Hatchlings and adult wild-type (*A*: *+/+*), Mack Super Snow (MSS) (*B*: *mss/mss*), and Mack Snow (*C*: *mss/+*) leopard geckos. We provide a close-up of the eye in the *Inset*.

Here, we focus on the leopard gecko (*Eublepharis macularius*; Fig. 1*A*), a small, nocturnal lizard found in the arid regions of Pakistan, south Afghanistan, and north India. The snout–vent length (SVL) of the hatchlings is 5.5 cm, and they more than double their size in adulthood (SVL = 13 cm) (3). Leopard geckos are one of the most widely kept species of reptiles in private colonies and over the past 50 y have been the subject of numerous biological studies that focus on their physiology, regeneration, temperature-dependent sex determination, behavior, and ontogeny [(4) and references within]. Numerous color and color pattern leopard gecko morphs exist that are often intermixed in the pet trade to generate spectacular new phenotypes (5). Very little work has been done to characterize them histologically and genetically, except for the Lemon Frost morph, which has been genetically characterized only recently (6). Leopard gecko hatchlings present transverse yellow and black bands, which gradually transform to irregular black spots during the first year of the animal’s life. Past research documented the ontogenetic changes of the hatchling to adult coloration by macroscopic imaging and grafting experiments (7), but without any histological analyses performed.

In this study, we first document the distribution of chromatophores in the skin of wild-type hatchlings and adults. We show that not only xanthophores and melanophores but also iridophores are present in the hatchling skin, very few of which persist in adults, contrary to what was previously suggested (8). Based on macroscopic time-lapse imaging of juvenile animals and further histological analyses, we describe the ontogenetic changes of the coloration pattern—from transversal bands to irregularly shaped black spots—as the animals mature. We also histologically and genetically characterize the MSS leopard gecko morph (Fig. 1 *B* and *C*). Hatchlings of this morph have a uniform black coloration that changes to black spots in adulthood. We find that these mutants lack both xanthophores and iridophores. With our mapping-by-sequencing analyses, we determine a 1.29 Mb genomic interval where the MSS mutation is situated. Within the interval, we identify a 13-nucleotide deletion that disrupts the protein coding sequence of the Paired Box 7 (*PAX7*) gene. PAX7 is a transcription factor required for xanthophore establishment in the zebrafish (9), as well as of xanthophores and leucophores in the medaka (10). Our single-cell transcriptomic analyses of embryonic dorsal skin from a leopard gecko show that PAX7 is expressed both in iridophores and xanthophores as they differentiate. Furthermore, our in situ hybridization experiments with *PMEL*, a marker expressed by all chromatophore progenitors, reveal that the bands fail to form in the MSS mutants. The above observations suggest that in leopard geckos, i) *PAX7* is associated with the differentiation of both iridophores and xanthophores, ii) the interactions of melanophores and iridophores give rise to the banded pattern of the hatchlings, and iii) melanophores can autonomously generate black spots in the postembryonic skin.

## Results

### Distribution of Chromatophores in the Leopard Gecko Skin.

Leopard gecko hatchlings carry transverse bands on their dorsal skin. A white band on the head is succeeded by a black band over the shoulder blades and then yellow and black bands alternate down to the base of the tail, which is covered by white and black bands (Fig. 1*A*). During the first year of the animal’s life, all bands fade away and black spots of variable sizes and shapes form (*SI Appendix*, Fig. S1). It has been suggested that the white bands on the head and tail of the hatchlings reflect the UV light, which is visible by predators, thus contributing to the antipredatory behavior of gaping and tail-waving that geckos deploy at this age. As the animals grow, they transition to a cryptic coloration that matches the escaping tactics of the adults (11).

**Fig. 2.**
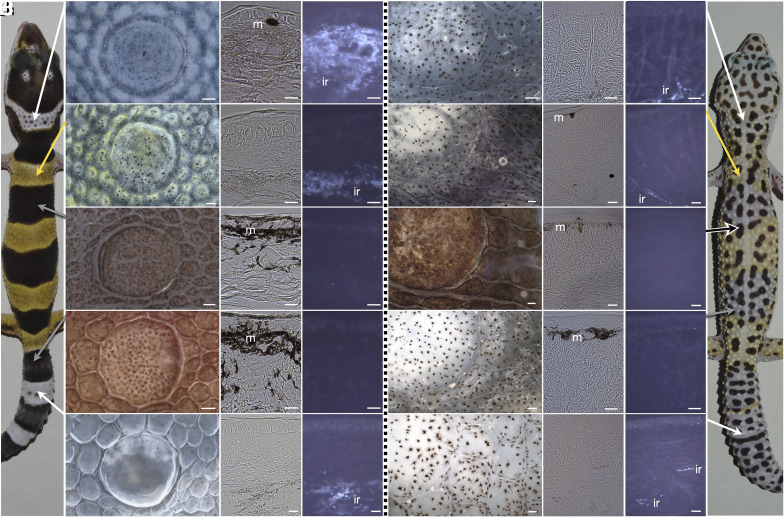
Chromatophore distribution in leopard gecko skin. (*A*–*E*) Hatchling skin from the white head band (*A*), a yellow body band (*B*), a black body band (*C*), a black tail band (*D*), and a white tail band (*E*). (*F*–*J*) Adult skin from the head (*F*), a former yellow band (*G*), a black body spot (*H*), a former black body band (*I*), and a former black tail band (*J*). Arrows point to the corresponding regions of a hatchling and adult wild-type leopard gecko. In all panels, the *Left* image corresponds to a single tubercule scale and the surrounding smaller ones, the *Middle* image to a transverse unstained paraffin section imaged with transmitted illumination, and the *Right* image to the same transverse section imaged with coaxial reflected illumination, where the beam of light is guided through the optics. m: melanophores, ir: iridophores. [Scale bars, 100 μm (*Right* image), 10 μm (*Middle* and *Left* images of *A*–*E*), and 20 μm (*Middle* and *Left* images of *F*–*J*).]

To document the ontogenetic changes of the dorsal skin coloration, we performed high-resolution imaging of the skin of hatchlings and adults and we used brightfield and transmission electron microscopy (TEM) on transversal skin sections (Fig. 2 and *SI Appendix*, Fig. S2). In the hatchling skin, the white head band is characterized by a dense network of iridophores covering the whole depth of the dermis and by dense collagen fibers (Fig. 2*A* and *SI Appendix*, Fig. S2*A*). Note that gecko iridophores appear highly dendritic (*SI Appendix*, Fig. S3*A*) and contain guanine crystals (*SI Appendix*, Fig. S4) of variable shapes and sizes (*SI Appendix*, Fig. S2*A*). The scarce dermal melanophores are contracted and situated just underneath the epidermis (Fig. 2*A*). The yellow bands on the body are populated by high numbers of xanthophores and a few contracted melanophores; iridophores can be seen deeper in the dermis (Fig. 2*B*). Most of the xanthophores observed by TEM contain vesicles with amorphous material, implying that it might be carotenoids that mainly contribute to the yellow coloration and less so pteridines usually located in vesicles with concentric lamellae (*SI Appendix*, Fig. S2*B*). Mass spectrometry analyses would be necessary to verify this point. We could detect only expanded melanophores in the dermis and epidermis of the black bands on the body and the tail (Fig. 2 *C* and *D* and *SI Appendix*, Fig. S2 *C* and *D*). In the white bands of the tail, iridophores are present, but in smaller numbers and deeper in the dermis compared to the white band of the head (Fig. 2*E*). Large bundles of collagen fibers are visible everywhere in the skin, especially in the thicker skin of the tail. Given their organization and regular spacing, these fibers could contribute to the light scattering, thus enhancing the brightness of the skin (12). No chromatophores were detected in the ventral skin, which owes its white color to the presence of dense collagen fibers (*SI Appendix*, Fig. S2*E*).

In the adult skin, the bands fade away and only black spots of variable sizes are clearly distinguishable (Fig. 1*A*). We sampled adult skin from the corresponding bands seen in hatchlings, and we observe that, throughout the body, the iridophores are less present in the dermis (Fig. 2 *F*, *G*, and *J*). As they maintain their dendritic aspect (*SI Appendix*, Fig. S3*B*), we assume that their number decreases and only a few iridophores persist to adulthood in the deeper layers of the dermis. Brightfield and TEM imaging confirms the continuous presence of xanthophores in the adult. Finally, melanophores persist near the epidermis; they are expanded in the black spots, where epidermal melanosomes can also be found (Fig. 2 *H* and *I* and *SI Appendix*, Fig. S5), and contracted in the surrounding light-colored areas lacking epidermal melanophores. A previous histological analysis of the leopard gecko adult skin (8) agrees with the localization of melanophores and xanthophores near the epidermis but failed to detect any iridophores. A likely explanation for this discrepancy is that in the adult skin, iridophores are scarce and situated deep in the dermis. We were able to detect them mainly by observing their reflectivity using coaxial reflected illumination and by careful observation of electron microscopy images taken at the deepest layers of the dermis.

### Ontogenetic Changes in the Leopard Gecko Skin.

**Fig. 3.**
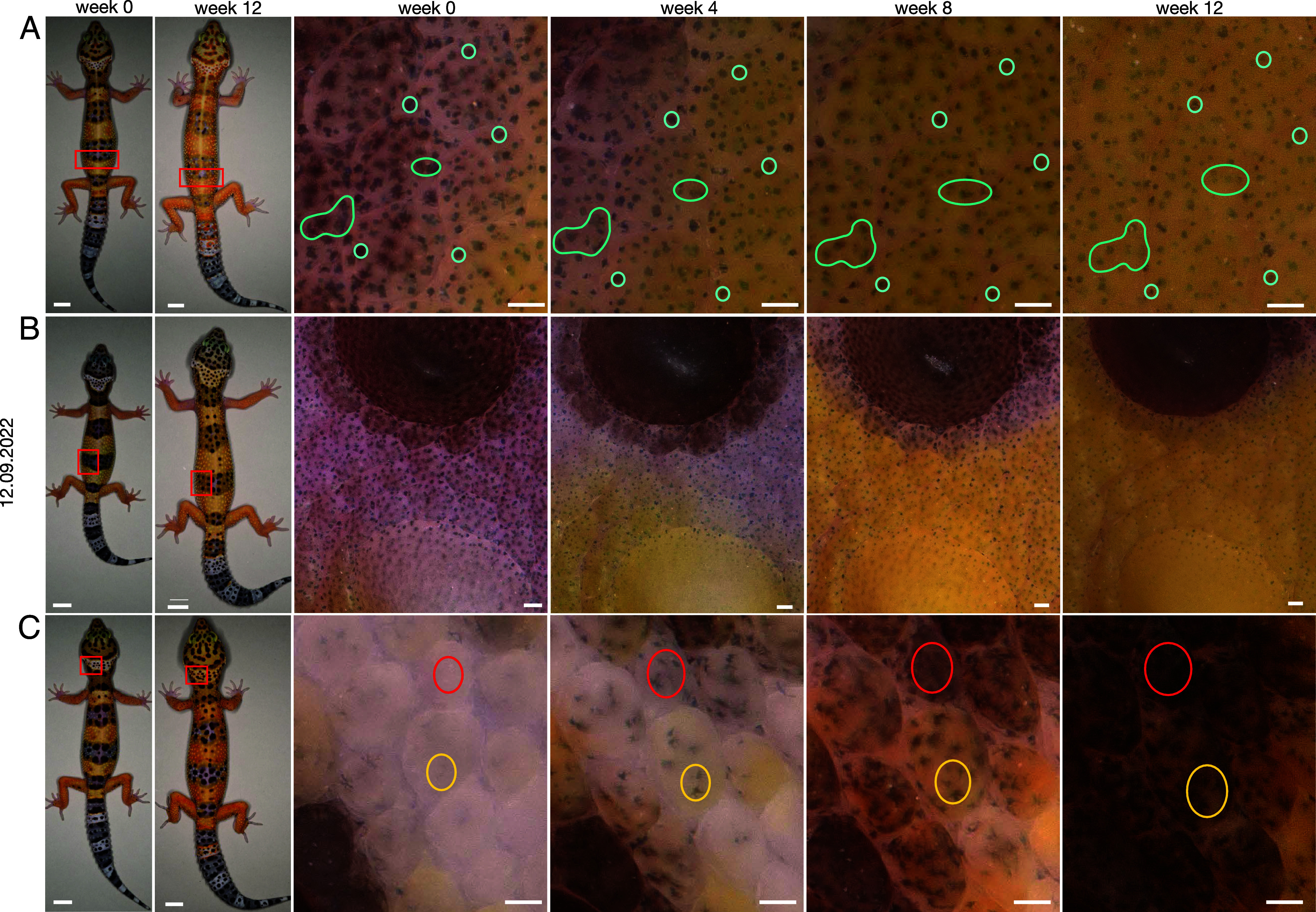
Ontogenetic color pattern transition. (*A*–*C*) Wild-type juveniles imaged over a period of 12 wk. For each animal, an overview image is shown at weeks 0 and 12. Magnified images of the regions in the red rectangles are provided for four time points (weeks 0, 4, 8, and 12). In *A*, we mark in cyan examples of melanophores that remain at the same position and gradually become contracted. In *B*, we observe that all melanophores gradually contract and the yellow coloration spreads. In *C*, the yellow circles mark melanophores that are already visible at week 0 and gradually expand. The red circles mark a region where no melanophores are visible at week 0 and gradually appear and expand in 12 wk. [Scale bars, 1 cm (overviews) and 100 μm (magnified areas).]

The transition from the hatchling to the adult pattern is characterized by the loss of the transversal black and yellow bands and the appearance of black spots. Although the skin of leopard geckos is not fully transparent, it is possible to distinguish the melanophores that are situated near the epidermis. We thus followed the ontogenetic transition of juvenile skin over a period of 12 wk and imaged selected regions at 4-wk intervals (Fig. 3). We noticed that melanophores are present in all regions, but they are contracted in the white and yellow bands and expanded in the black bands and spots. The reduced transparency of the skin in the black regions also suggests the presence of epidermal melanophores.

Time-lapse imaging of the black bands revealed that black spots form as the melanophores in the surrounding areas contract and the epidermal melanophores disappear making the surrounding skin light-colored (Fig. 3 *A* and *B* and *SI Appendix*, Fig. S5). At the same time, the xanthophores populate the vacant regions, possibly by migration and division from the nearby yellow bands. The black spots in the white and yellow bands can form in three different ways: i) the migration of melanophores, ii) the proliferation of existing melanophores, or iii) the expansion of existing melanophores. In the skin region that we followed (Fig. 3*C*), no melanophore migration was evident at the top layers of the dermis, although we cannot exclude that it does take place given that the leopard gecko skin is not fully transparent. The visible melanophores remained at the same position during the 3-mo period, but their state changed from contracted to expanded. At the same time, the transparency of the skin decreased probably due to the appearance of epidermal melanophores. We thus assume that melanophore division and state transition are the main drivers of black spots formation on the white and yellow bands. Similar were the conclusions of Whimster (7), who also followed the ontogenetic modifications of the leopard gecko skin. In particular, he performed grafting experiments where he rotated full-depth skin grafts by 180°; although black spots formed across the graft boundaries, no melanophore migration was observed.

In the zebrafish, melanophores have limited capacity to spread in the skin when the density of neighboring chromatophores is high, but they can move freely when this density is decreased by ablation (13). If melanophore migration does occur in the leopard gecko skin, it would imply that their environment is changing in the transition from hatchling to adult. Regarding division, the proliferative ability of zebrafish melanophores diminishes after differentiation (14), which would be in contrast with our suggestion. With the development of gene-editing protocols in reptiles (15, 16), cell tracing experiments might become possible in the future to elucidate this point.

### Thyroid Hormonal Sensitivity of the Leopard Gecko Skin.

In metamorphic species, such as frogs, thyroid hormones (TH) play an important role in the transition from larvae to adult (17). They have also been shown to regulate the development of the adult pigment pattern in the zebrafish (18, 19). Indeed, hypothyroid zebrafish have a reduced number of xanthophores and an increased number of melanophores, whereas the reverse is observed in hyperthyroid animals (18). In leopard geckos, TH receptors A and B (THRA and THRB) are present in the epidermis and higher expression levels coincide with the shedding cycle of the animals (20).

We hypothesized that the embryonic leopard gecko dermis is sensitive to TH and that it gradually loses this sensitivity in the transition from juveniles to adults, thus affecting the coloration pattern. We performed semiquantitative PCR of the *THRA* and *THRB* transcripts on adult and juvenile whole skin (dermis and epidermis), as well as disassociated dermis and epidermis from regions with different coloration (*SI Appendix*, Fig. S6). Both receptors were expressed in all the samples tested suggesting that the sensitivity of the skin to TH remains the same as the animal grows. These observations do not exclude the possibility that the level of TH varies in the juvenile and the adult. For example, it has been shown in clownfish that the faster formation of white bars, composed of iridophores, in certain juveniles is associated with higher levels of TH (21). Obviously, we cannot exclude that other factors play a role in the ontogenetic changes described above, namely the reduction in iridophore numbers and the formation of spots by the melanophores.

### Chromatophores in the Regenerated Gecko Skin.

When the leopard gecko skin regenerates, chromatophores migrate from the adjacent intact skin, as it has been shown for the regenerated tail (22, 23) and dorsal skin (7, 24, 25). On the regenerated tail, melanophores directly form black spots as in the adult dorsal skin, rather than yellow and black bands as seen in hatchlings (*SI Appendix*, Fig. S7 *A*–*C*). Note that spots form even on the regenerated tail of juveniles, despite the presence of bands on their body (*SI Appendix*, Fig. S7*C*). Scarce spots of xanthophores can be observed in some cases (*SI Appendix*, Fig. S7*C*) and contracted melanophores are found in the white space between the black spots (*SI Appendix*, Fig. S7*D*). Histological sections confirmed the absence of iridophores in the regenerated skin (*SI Appendix*, Fig. S7 *E* and *F*). These observations suggest that in the absence of iridophores, melanophores directly and autonomously form spots on the regenerated tail. The presence of xanthophores does not affect the pattern.

### Skin Color Pattern of the MSS Geckos.

Leopard geckos homozygous for the MSS mutation, emerged in the breeding stock in 2004. They are characterized by the uniformly black coloration of the hatchlings and the black spots covering the body of the adults (Fig. 1*B* and *SI Appendix*, Fig. S8). At no moment of their development is white and yellow coloration visible. On the contrary, Mack Snow leopard geckos, which are heterozygous for the mutation, hatch with white and black bands. The white bands of these hatchlings are populated by iridophores (*SI Appendix*, Fig. S9). It is in the first 10 to 12 wk after hatching that the white bands of their body acquire the yellow pigmentation. The heterozygous animals subsequently go through the ontogenetic changes as the wild-type ones (Fig. 1*C*). Late-stage wild-type embryos (50 d post oviposition for a 60-d incubation) also present white and black bands and the white bands gradually turn yellow before hatching. We thus assume that the appearance of yellow pigmentation is delayed in Mack Snow (heterozygous) hatchlings, compared to the wild type.

**Fig. 4.**
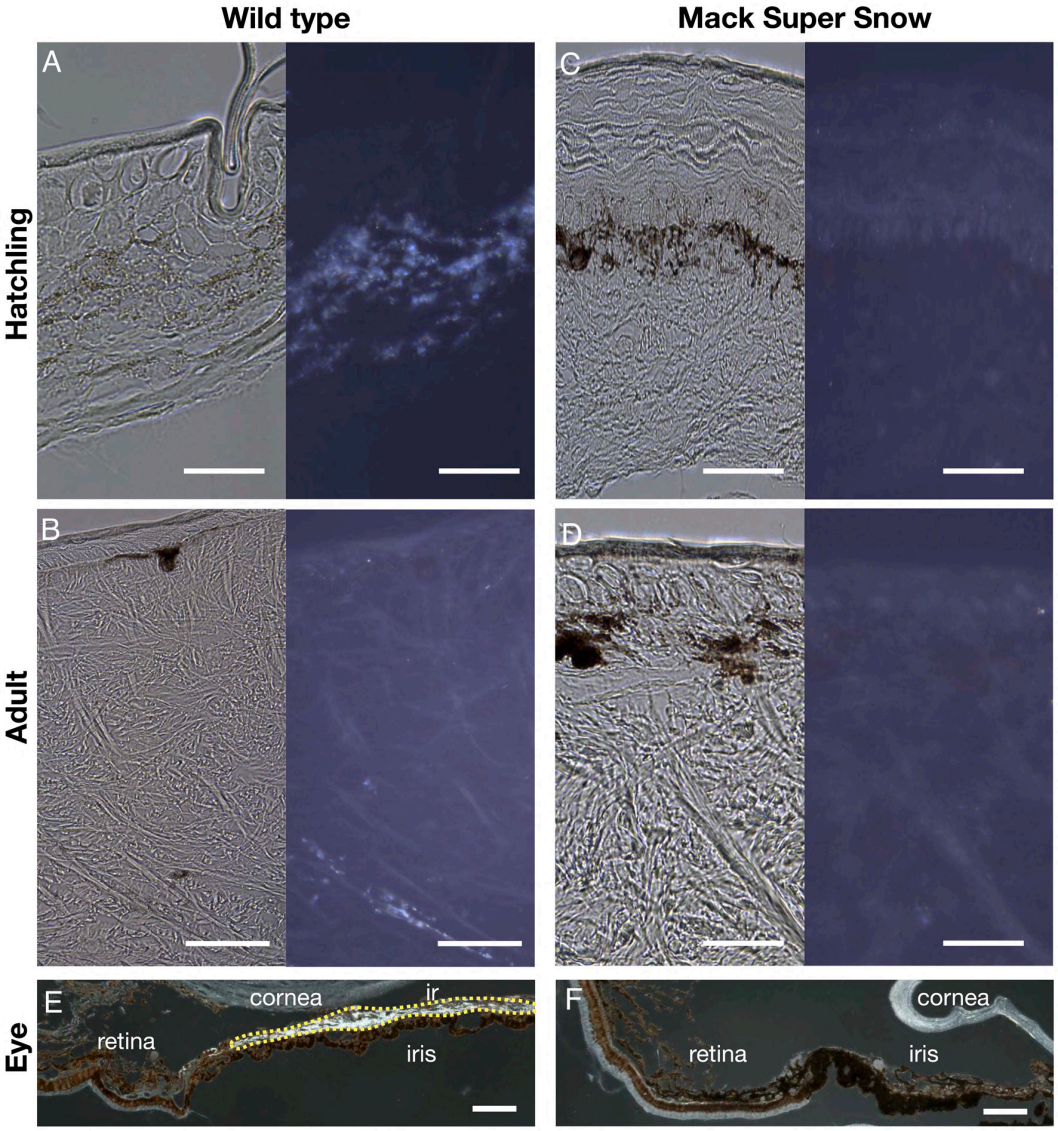
MSS leopard geckos lack iridophores. Comparison of transverse paraffin sections of the hatchling and adult wild type (*A* and *B*) and MSS (*C* and *D*) skin imaged with transmitted and reflected coaxial illumination. The hatchling MSS dorsal skin is homogeneously colored black. For *D*, we sampled a black spot. No iridophore reflection is observed in both MSS samples. (*E*) Transverse section through the retina and the iris of a wild-type leopard gecko, where the iridophores’ reflectance is visible (yellow dotted line). (*F*) Transverse section through the retina and the iris of a MSS leopard gecko lacking iridophores. [Scale bars, 20 μm (*A*–*D*) and 100 μm (*E* and *F*).]

Histological analyses of the skin of MSS animals revealed that only melanophores are present both in hatchlings and in adults (Fig. 4 *A*–*D* and *SI Appendix*, Fig. S2*F*), confirming the autonomous capacity of melanophores to generate black spots in the postembryonic skin. Wild-type eyes are characterized by black strikes on a silver background with an elliptical black pupil, whereas the eyes of MSS geckos appear uniformly black (Fig. 1 *A* and *B*). Sagittal sections of the eyes confirmed that the silver background in the wild type is due to the presence of iridophores in the iris, which are absent from the iris of MSS (Fig. 4 *E* and *F*). Measurements of the black spots in adult wild-type and MSS animals showed that their area is variable among individuals, but the range is similar between the two morphs (*SI Appendix*, Fig. S10).

### Mapping of the Causative Variant for the MSS Geckos.

**Fig. 5.**
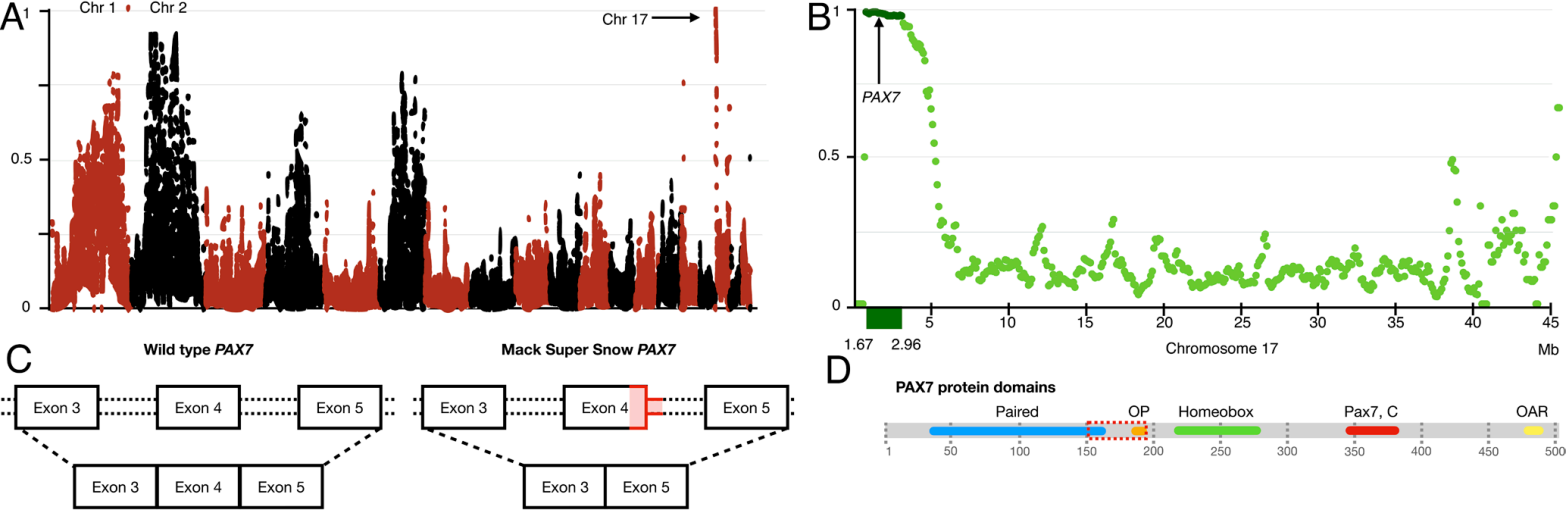
Mapping the *MSS* locus and the *PAX7* structure. (*A*) Proportion (*y* axis) of quality-filtered SNP/MNPs cosegregating with the *MSS* locus in the four genome libraries compared to informative quality-filtered parental variants (homozygous in the *mss/mss* father and heterozygous in the *mss/+* females). Proportions are calculated with a 1 Mb sliding window and a step of 100 Kb. Chromosomes (alternatingly colored black and red) are ordered from longest to shortest, and Chromosomes 1, 2, and 17 are pointed at. (*B*) Close-up of the genome interval harboring the *MSS* locus. Proportion (*y* axis) of quality-filtered SNP/MNPs cosegregating with the *MSS* locus in the four genome libraries compared to informative quality-filtered parental variants (homozygous in the *mss*/*mss* father and heterozygous in the *mss*/+ females). The proportion was calculated in overlapping windows of 1 Mb with a step of 100 Kb. Dark green dots correspond to the 1.29 Mb region with the highest proportion of cosegregating variants. *PAX7* is in this interval. (*C*) The 13-nucleotide deletion in MSS (in red) spans the last six nucleotides of exon 4 and the first seven nucleotides of intron 4. As a result, exon 4 is expected to be absent from the amplified MSS transcript. (*D*) InterProScan domains of the wild type and MSS PAX7, along with the Octapeptide (OP, in orange) that was not predicted by InterProScan. In MSS PAX7, the region in the dashed red rectangle is missing. Thus, the paired domain is shorter, the OP is absent and the distance between the paired domain and the homeodomain is reduced.

Test crosses confirmed that a single locus mutation is responsible for the MSS morph and the wild-type allele presents incomplete dominance over the MSS one, as attested by the heterozygous phenotype (Fig. 1*C*). We performed mapping-by-sequencing analyses to retrieve the causative variant of the MSS leopard gecko morph, as previously described (26). In short, we crossed a homozygous MSS male (*mss*/*mss* genotype) with two heterozygous females (*mss*/+) to obtain homozygous and heterozygous offspring for the causative allele. We sequenced four genomic DNA libraries, one for the homozygous male, one for the two heterozygous females, one for the pool of homozygous offspring and one for the pool of heterozygous offspring (*SI Appendix*, Table S1; NCBI accession number PRJNA983248) (27). We aligned each library separately to the leopard gecko genome [(28); GCF_028583425.1] and searched for nucleotide polymorphisms (SNPs and MNPs) cosegregating with the MSS genotype in nonrepetitive elements (26). We thus identified an interval of 1.29 Mb at the beginning of Chromosome 17 (NC_072806.1 from 1.67 to 2.96 Mb; Fig. 5 *A* and *B*). There are 2,804 cosegregating SNPs/MNPs with a density of 2,165 variants/Mb. A 1-Mb window on Chromosome 1 contains a single SNP that randomly cosegregates with the causative allele, and Chromosome 2 shows a relatively high cosegregation (*SI Appendix*, Fig. S11), but not as high as at the beginning of Chromosome 17 and the density of cosegregating SNPs is lower than in Chromosome 17.

Based on the NCBI RefSeq annotation of the leopard gecko genome, there are 37 annotated genes in the interval (*SI Appendix*, Table S2). We looked for variants (polymorphisms or indels) that cosegregate with the MSS genotype and result in nonsynonymous mutations in the coding sequence of the leopard gecko genes. Using this approach, we identified single- and multinucleotide polymorphisms within the coding sequence of 10 genes that result in amino acid polymorphisms. A 6-nucleotide deletion in the coding sequence of the Chloride transport protein 6 (*CLCN6*) gene removes two amino acids from the protein without affecting its structure, according to InterProScan analyses (29). On the other hand, a 13-nucleotide deletion disrupts the coding sequence of the Paired box 7 (*PAX7*) gene (Chromosome 17; position 1,828,551; *SI Appendix*, Fig. S12). It is located at the boundary of exon 4 and intron 4 and results in the removal of six nucleotides from the coding sequence (Fig. 5*C*). We designed primers flanking the 13-nucleotide deletion in the MSS morph. We genotyped the 29 individuals used for the family mapping (13 *mss*/*mss*, 16 *mss*/+) and 148 individuals from 24 different lineages (15 *mss*/*mss*, 45 *mss*/+, and 88 +/+) and confirmed that the deletion is fixed in MSS and Mack Snow individuals (*SI Appendix*, Tables S3 and S4) and absent from +/+ animals.

### Differential Expression of *PAX7* Isoforms in the Wild Type and MSS.

Mammals and reptiles have nine members of the paired box family, from PAX1 to PAX9. The paired box proteins are classified in groups depending on the protein domains they contain, with all of them presenting an N-terminal DNA-binding paired domain (30). Group III includes PAX3 and PAX7, which are paralogs originating from the same ancestral gene (31). In addition to the paired domain, they have a DNA-binding homeodomain and a conserved octapeptide region found in most PAX genes, which is located between the two other domains. The octapeptide domain includes a conserved TN8TCCT DNA motif, with N8 being any combination of eight nucleotides (32). Based on InterProScan (29) predictions, the wild-type leopard gecko PAX7 has the following domains: the paired domain (34-163 aa), the homeodomain (215–279 aa), the PAX7 C-terminal domain (344–383 aa), and the OAR domain (477–489 aa) (Fig. 5*D*). The octapeptide (OP; 186–193 aa) is not predicted, despite that it has the same sequence as in mice [HSIDGILG; (33)].

Mutations at the canonical donor splicing sites usually lead to single exon skipping (34), so we expect exon 4 to be missing from MSS *PAX7* transcripts (Fig. 5*C*). We designed primers to amplify the *PAX7* isoforms (*SI Appendix*, Fig. S13*A*) and found that four different *PAX7* isoforms are expressed in wild-type animals, three of which are functional (WT1-3) and one not (WT4), due to an early STOP codon (*SI Appendix*, Fig. S13*B*). In MSS, we amplified six *PAX7* isoforms, three lack exon 4 as expected (MSS1-3) and three maintain exon 4 but have a 118-nucleotides insertion (MSS4-5), which corresponds to the beginning of intron 4 (*SI Appendix*, Fig. S13 *B*, *C*). Only MSS1 and MSS3 are possibly functional, despite the deletion of exon 4, because no frameshift is introduced. The resulting PAX7 protein is reduced from 503 to 458 aa and, compared to the wild type, amino acids 151 to 195 are missing. This deletion shortens the paired domain, although it is still predicted by InterProScan, and the octapeptide is missing. In mice, it has been demonstrated that the region between amino acids 27 and 208 mediates the interaction with PAX3- and PAX7-binding protein 1 (PAXBP1), which subsequently links PAX3 and PAX7 to the histone methylation machinery (35). Hence, the deletion is likely to affect this interaction. Finally, the 45-amino acid deletion may affect the entire 3D conformation of PAX7, as the distance between the paired domain and the homeodomain is reduced, making it nonfunctional even if these domains are not affected.

We performed semiquantitative amplification of the main *PAX7* isoforms on samples from wild-type and MSS embryos (*SI Appendix*, Fig. S14). For both morphs, we sampled the dorsal skin and a body part composed of mesenchymal tissues (mainly muscles and bones, but no skin) from developmental stage 35 embryos (36). Both wild-type samples mainly express the functional isoforms WT1-3. In the dorsal skin of the MSS, we only observe faint expression of MSS6, which is probably not functional. If other isoforms are produced, their expression level is below the sensitivity of our amplification. In the body part of the MSS, we mainly detect the expression of MSS1 and MSS3, which are probably partially functional despite the exon 4 deletion. Based on the above, we conclude that i) no functional PAX7 is produced in the skin of MSS geckos and ii) possibly functional isoforms are expressed in the mesenchymal tissues, ensuring the proper development of muscles.

### PAX7 Is Required in the Fate Termination of Xanthophores and Iridophores.

In mammals, PAX7 plays an important role in neural crest development and the determination of the myogenic cell lineage (37). It is also expressed in muscle satellite cells, that support muscle growth and regeneration. In humans, deletion of the OAR domain results in a neuromuscular deficient phenotype (38); whereas in mice, PAX7 mutations mainly affect the musculature (39). In zebrafish, Pax7 is involved in but not required for myogenesis (40). On the other hand, Pax7 is necessary for the proper migration and maturation of the xanthophore lineage cells (41) in the same species. Due to an extra whole genome duplication in teleost, zebrafish have two copies of *pax7* (a and b). Zebrafish double mutants for *pax7a* and *pax7b* produce a reduced number of xanthophore precursor cells (i.e., xanthoblasts) and lack differentiated xanthophores and yellow pigmentation (9). Indeed, low levels of expression of typical xanthophore lineage markers, such as *gch2* (GTP cyclohydrolase 2), *xdh* (xanthine dehydrogenase), and *csf1r* (colony stimulating factor 1 receptor, a), were detected in embryos of these mutants. In adult double mutants, the number of iridophores is reduced and the melanophores form aggregates, instead of black stripes. In medaka, a *pax7a* mutant presents defects in the differentiation of leucophores and xanthophores, with no effect in the other chromatophores (10).

Little information is available on the chromatophore differentiation process during embryogenesis in reptiles and the molecular signaling implicated (42, 43). To verify whether *PAX7* is involved, we performed single-cell RNA sequencing on dissociated cells from the dorsal skin of a wild-type embryo at developmental stage 40 (GEO accession GSE264342 (44); Dataset S1). At this stage, faint black bands are visible for the first time, but no other pigmentation is present. After quality check and filtering, we kept 2,738 cells, which were clustered using the Louvain algorithm with a resolution of 0.8. The cell types were identified based on mouse skin (45), human tissue (46), and zebrafish chromatophore (19) markers (*SI Appendix*, Fig. S15).

**Fig. 6.**
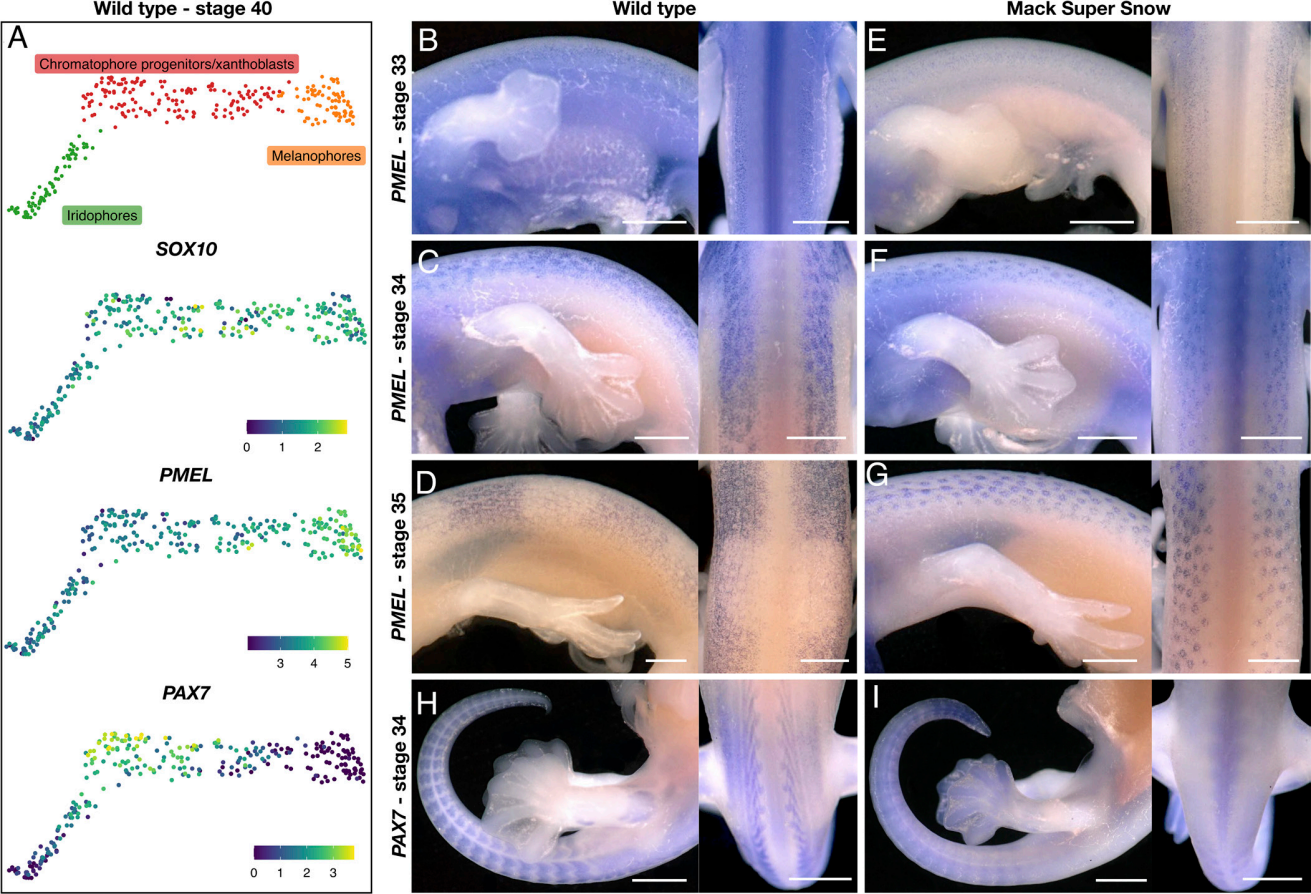
Single-cell transcriptomic analysis of developing chromatophores and their spatial distribution on leopard gecko embryos. (*A*) Cluster identification within chromatophores and UMAP representation of the expression of selected chromatophore differentiation markers. (*B*–*D*) Wild-type embryos at stages 33, 34, and 35. The homogenous *PMEL* expression changes to the banded pattern of the hatchling. (*E*–*G*) MSS embryos at stages 33, 34, and 35. The *PMEL* expression remains homogeneous, and labeled cells mainly aggregate around the tubercles. (*H* and *I*) Wild-type and MSS embryos at stage 34 labeled with a *PAX7* probe. The skeletal muscles are labeled in both samples, although labeling is fainter on the MSS embryo. (Scale bar, 100 μm.)

Most chromatophores (97.7%) express *SOX10* (Fig. 6*A*), as do the radial glial cells (94.7%), attesting their neural-crest origin (*SI Appendix*, Fig. S15*B*). *PMEL*, which is essential for the formation of fibrillar sheets in melanophores (47), is expressed by all chromatophores and at higher levels by the melanophores (Fig. 6*A*). The expression profile of *pmela* is similar in the zebrafish, based on single-cell transcriptomic data (19). *MITF* is also expressed by all, but less so in iridophores (*SI Appendix*, Fig. S15*B*). This is in agreement with our previous finding, that *MITF* is involved in the differentiation of melanophores and xanthophores in the leucistic Texas Rat snake (42). In zebrafish, *mitfa* is also expressed in progenitor cells and differentiated melanophores.

The first chromatophore cluster specifically expresses *OCA2*, encoding the p protein which is involved in melanogenesis, and *CORIN*, that specifies coat color in mice by suppressing *agouti* (48). The expression of *TYR*, *TYRP1*, and *DCT*, all of which are involved in melanogenesis, is less specific yet stronger in this cluster, suggesting that these are differentiating and mature melanophores (*SI Appendix*, Fig. S15 *B* and *C*). The second cluster specifically and strongly expresses several well-established iridophore markers from zebrafish studies, such as *TFEC*, *ALX4*, and *PNP* (*SI Appendix*, Fig. S15 *B* and *C*) (19), so we annotated them as differentiating iridophores. The third chromatophore population shows higher levels of *SOX10* expression (Fig. 6*A*), implying that these neural-crest derived cells are at an earlier stage of differentiation, as well as *PTSP*-*like*, which encodes an enzyme in the pteridine synthesis pathway (*SI Appendix*, Fig. S15 *B* and *C*). We thus assume that this cluster either groups chromatophore progenitor cells and xanthoblasts or comprises only xanthoblasts. In both cases, it seems that xanthophores are at an earlier stage of differentiation than the iridophores and the melanophores.

*PAX7* is strongly expressed in the progenitors/xanthoblasts, and at a lower level in iridophores (Fig. 6*A*). As expected, expression is also seen in the muscle cells (*SI Appendix*, Fig. S15*B*). In zebrafish, *pax7a* is expressed in iridophores and both *pax7a* and *pax7b* are expressed in xanthophores (19). So, the expression profile of the single *PAX7* copy in leopard geckos resembles the one of *pax7a* in zebrafish, and the phenotype of the MSS mutants suggests that *PAX7* is necessary for the differentiation of both iridophores and xanthophores. We observe that these two chromatophores also share the expression of *LTK* (*SI Appendix*, Fig. S15*B*), required for the development of iridophores in zebrafish (49). Such functional diversification of coloration genes has been previously reported; for example in axolotl, *Ltk* mutants lack all iridophores and have fewer xanthophores (50).

### Pattern Formation in Wild-Type and MSS Geckos.

Given the high levels of *PMEL* expression by all chromatophores, we used a *PMEL* probe to track the pattern formation during the leopard gecko development by performing whole-mount in situ hybridizations (WISH). In the wild type, *PMEL* is only expressed by a few skin cells at each side of the vertebral column at stage 33 (Fig. 6*B*). At stage 34, a uniform expression can be seen bilaterally, above the forelimbs and on the tail (Fig. 6*C*). At stage 35, the density of the labeled cells is greater at the future black bands (Fig. 6*D*). Note that these bands form independently at the right and left dorsal side of the body and they will eventually merge as the skin fully develops over the vertebral column. Furthermore, the labeled chromatophore precursors aggregate around the developing tubercles, which are the largest scales seen on the dorsal skin.

In MSS embryos, *PMEL* is similarly expressed in stages 33 and 34 (Fig. 6 *E* and *F*). At stage 35, a greater concentration of labeled cells is seen around the developing tubercles, but no transverse bands are visible, as in the wild type (Fig. 6*G*). We assume that in MSS geckos, iridophores and xanthophores do not differentiate, and in their absence, the melanophores maintain a homogenous distribution, as seen in the hatchlings. We also performed WISH at stage 34 with a *PAX7* probe that spans the intact exons 5 to 9. In both wild-type and MSS animals, only the developing muscles were labeled. Although quantification is not accurate with WISH, we do observe fainter *PAX7* expression on the MSS embryo. The expression levels of *PAX7* in the skin might be below the sensitivity threshold of WISH experiments or it might be expressed at a different developmental stage. We also performed WISH with probes against known xanthophore (*GCH1*, *XDH*) and iridophore (*TFEC*) markers, as well as markers selected for their specificity and high expression level from our single-cell data (*CALM1* for xanthophores, *CALM2* for iridophores, and *CRIP1* for melanophores) (*SI Appendix*, Fig. S16). No signal was observed for *GCH1, XDH, TFEC,* and *CALM2*. This is expected for *GCH1* and *XDH* as they saw no expression with the single-cell data. For the remaining genes, the lack of signal is either because these genes are not expressed at the stages when WISH is performed (stage 35), or because their expression level is very low. With the *CALM1* probe, we observe faint expression at a location where the future yellow band will form on the hatchling. This result should be taken with caution as the staining is very faint. Regarding *CRIP1*, the muscle cells seem stained, but we did not observe any staining in the skin. Note that it is not possible to run WISH experiments at the stage when the single-cell RNA sequencing was done (stage 40), because the keratinization of the skin is advanced, and black pigmentation is already visible.

## Discussion

Squamates do not undergo metamorphosis, yet the coloration pattern of the leopard gecko changes as the animal develops, grows, and sexually matures. Based on our whole mount in situ hybridization experiments, we hypothesize that the chromatophore progenitors spread homogeneously on the dorsal embryonic skin while they differentiate from the neural crest cells. We conclude from the single-cell transcriptomic analyses that melanophores and iridophores are the first to take up their identity. We base this assumption on the expression profiles of these two cell types, which include terminal differentiation markers, as well as on the observation that during development, melanophores are the first to produce melanin, followed by iridophores that accumulate guanine crystals, and finally xanthophores that become yellow just before hatching. It is likely that the early-stage interactions of differentiating melanophores and iridophores are responsible for the establishment of the transverse bands. Support for this hypothesis comes from the developing MSS embryos, that lack both iridophores and xanthophores. In these embryos, the bands fail to form, and the hatchlings have a uniform black coloration. To confirm this scenario, we would need to i) perform single-cell transcriptomic analyses at earlier and later developmental stages to understand the dynamics of the chromatophores differentiation, ii) identify species-specific and chromatophore-specific markers, and iii) use these markers for whole mount in situ hybridizations to visualize the spatial distribution of the maturing chromatophores during the patterning process. From our transcriptomic study, we also conclude that *PAX7* is required for the differentiation of both iridophores and xanthophores, explaining their absence from the MSS animals, which carry a mutated copy of this gene. This observation puts forward the diversification of the coloration genes, as they take up different functions among and within vertebrate lineages.

In the postembryonic skin, the behavior of the chromatophores changes. Iridophores perish, xanthophores expand, and melanophores form irregularly shaped black spots (*SI Appendix*, Fig. S17), similarly to the *shady* phenotype of the zebrafish (51). We provide two lines of evidence for the capacity of melanophores to autonomously form black spots in this context. The first is the coloration of the regenerated tail, where melanophores directly aggregate to form spots. The second is the transition from the uniform black coloration of the MSS hatchlings to black spots in the adult. As our investigation of the expression profile of the TH receptors was not conclusive, the factors triggering the transition from the hatchling to the adult pattern remain unknown. The presence or absence of xanthophores seems to have little effect on the spot formation in the postembryonic skin. On the other hand, evidence on the importance of the iridophores–melanophores interactions comes from the Super Lemon Frost leopard gecko morph. These animals maintain iridophores to adulthood and are prone to the development of iridophoroma, a tumor of iridophores (52), due to a mutation in *SPINT1*, which is a tumor suppressor (6). The transversal black bands remain evident in adult Super Lemon Frost animals and the melanophores seem to form small circular aggregates, rather than irregular spots. These spots are similar to the ones observed on the neck white band of the wild-type juvenile, where a dense network of iridophores is also present, and resemble the *pfeffer* phenotype of the zebrafish (51). These observations support the inhibitory interactions between iridophores and melanophores during development and after hatching and the tendency of melanophores to aggregate in the postembryonic skin. Overall, our study provides insights into the regulatory mechanisms governing skin coloration patterning in leopard geckos and improves our understanding of color pattern formation in reptiles.

## Materials and Methods

### Experimental Model.

Leopard geckos were housed and bred at the LANE animal facility running under veterinary cantonal permit no. 1008. The individuals were sampled and imaged following Swiss law regulations and under experimentation permit GE24/33145.

### Imaging.

Animals were imaged under anesthesia using R^2^OBBIE (53), an automated image acquisition system, and the VHX-7000 (Keyence). Skin samples were imaged using the VHX-7000 (Keyence). The area of spots was measured on images of four wild-type and four MSS using the “Analyse particles” function in Fiji.

### Histology.

Skin and eyes were fixed in 4% paraformaldehyde and dehydrated in ethanol before embedding in paraffin blocks. Seven-micrometer microtome sections were deparaffinized and directly imaged with VHX-7000 (Keyence). Skin samples were fixed in 4% paraformaldehyde and permeabilized in PBS/Triton 0.5%. The nuclei were stained with ToPro3 (ThermoFisher, T3605) at 1:2,500 and the samples were cleared with RapiClear 1.52 (SunJin Lab, RC152001) before imaging with an SP8DIVE confocal microscope.

### TEM.

Skin pieces of 1 mm^2^ were fixed and sectioned as previously described (26). Micrographs were taken with a transmission electron microscope Philips CM100 (Thermo Fisher Scientific) at an acceleration voltage of 80 kV with a TVIPS TemCam-F416 digital camera (TVIPS GmbH). Large montage alignments were performed using Blendmont command-line from IMOD software (54).

### RAMAN.

Raman spectroscopy was performed on a 10 μm cryosection of neck white skin from a wild-type hatchling. The chemical structure of a target molecule is determined by measuring the energy shift of an incident laser light after its interaction with the molecule. The spectrum was collected on a Horiba Xplora spectrometer. The excitation source was a solid-state laser at 785 nm. The spectrum (black line) was matched against the KnowItAll database (Bio-Rad) of confirmed spectra and clearly supports the presence of guanine (red line) in the reflecting platelets of the iridophores.

### Crosses and Deep Sequencing of MSS Individuals.

We crossed a MSS male (*mss*/*mss* genotype) and two wild-type females with a heterozygous (*mss*/*+*) genotype to obtain homozygous and heterozygous offspring. Genomic DNA was extracted from the parents and the hatchlings using the QIAGEN DNeasy Blood and Tissue kit (69504) following the manufacturer instructions. We prepared and sequenced separately three pools of DNA samples from: i) 12 *mss*/*mss* offspring, ii) 14 *mss*/+ offspring, and iii) the two parental females in equimolar concentrations, as well as the parental male. We sequenced the TruSeq DNA PCR Free libraries using an Illumina HiSeqX instrument, producing 151 bp paired-end reads (Macrogen). We obtained 189 to 199 million of paired-end reads per library and checked data quality and the absence of adapters with FASTQC. We performed a quality filtering with sickle v1.33 (55). We retained between 174 and 183 million reads after this step, which corresponds to a 23.9 to 25.2× average coverage for a 2.2 Gb genome.

### Variant Calling.

We aligned the four genomic libraries of the parents and the offspring against the leopard gecko genome (28) using bwa v0.7.17 (56) with default parameters in mem mode. We converted the output SAM files into BAM, removed duplicates using the fixmate mode with the -m flag and the markdup mode with the -r flag and sorted them out by their leftmost coordinates with SAMtools v1.9 (57). We identified genomic variants and retrieved the genomic interval where the *MSS* locus is located as previously described (26) using VCFtools v0.1.16 (58) and Platypus v0.8.1 (59). We considered any scaffold having regions with a minimum of 100 consecutive cosegregating polymorphisms.

### *PAX7* Amplification and Cloning.

We extracted genomic DNA using the DNeasy Blood & Tissue Kit and genotyped them for the 13-nucleotide deletion identified by deep-sequencing. We extracted mRNA with the Direct-zol RNA Miniprep kit (Zymo Research, R2081) and prepared complementary DNA with PrimeScript™ Reverse Transcriptase (TakaraBio, RR047A). We then amplified and sequenced the *PAX7* transcript in MSS and wild-type individuals. The primers for the *PAX7* variant verification on cDNA and gDNA samples are provided in *SI Appendix*, Table S5.

The amplification products of the primers Pax7_18B-F and Pax7_1087-R were purified with the ChargeSwitch PCR Clean-Up Kit (ThermoFischer, CS12000) and then ligated in the vector PCRII using the TA Cloning Kit (ThermoFischer, K2060-01). Vectors were transformed in One Shot™ TOP10F’ Chemically Competent *Escherichia coli* by heat shock at 42 °C, then plated on IPTG (0.05 mM), X-Gal (0.08 mg/mL), and ampicillin (0.1 mg/mL) LB agar petri dishes. White transformants were amplified in LB High salt medium using the QIAprep Spin Miniprep Kit (27106). Clones were then Sanger sequenced using SP6 and T7 primers.

### Semiquantitative PCR.

We extracted mRNA and prepared complementary DNA as described above. For the THR amplification, we started with 200 ng of mRNA as measured with the Qubit Fluorometer. For the *PAX7* amplification, we started with 400 ng of mRNA. Due to the low expression levels of some transcripts, we performed 35 amplification cycles, and the annealing temperature was set to 58 °C. We used the FastStart Taq-DNA-Polymerase (Merck, 12032902001). The housekeeping gene Delta-aminolevulinate synthase 1 (*ALAS1*) was the reference.

### scRNA-seq Sample Preparation and Sequencing.

E35 wild-type leopard gecko embryo dorsal skin was isolated and dissociated in a single-cell suspension according to a protocol used for chicken embryos (60). The transcriptome was examined using the Chromium Next GEM Single Cell 3′ Reagent Kits v3.1 (10× Genomics) according to the manufacturer's instructions. Sequencing was performed in a HiSeq X Ten (Illumina) (Read1: 28 cycles; Read2: 90 cycles; i7 index: 10 cycles; i5 index: 10 cycles) at an average depth of 50,000 reads/cell. The sequenced libraries were demultiplexed and converted to FASTQ files using the mkfastq function from Cell Ranger v6.1.2 (10× Genomics). Reads were aligned to the *E. macularius* NCBI reference genome (GCF_028583425.1, MPM_Emac_v1.0) using the Cell Ranger (v7.2.0) count function with default parameters. The reference was generated with the Cell Ranger mkref function after selecting the recommended gene biotypes from the genome annotation with the mkgtf function. The median number of unique molecular identifiers (UMIs) per cell was 11,480, with a median of 3,340 genes for the 4,324 cells detected by Cell Ranger.

### Analysis of the scRNA-seq Data.

Most computational analyses of the resulting UMI filtered count matrix were performed using the R package Seurat (v4.2.0) (61). Doublets were detected with scDblFinder v1.12.0 (62), considering default parameters. Cells were subjected to a quality control step, keeping those detected as singlets, with less than 10% of UMIs assigned to mitochondrial genes (prefix “BW219-”), less than 30% of UMIs assigned to ribosomal genes, expressing more than 1,000 genes and with more than 2,000 detected UMIs. These thresholds were chosen upon visual inspection of distributions. After filtering, we kept 2,738 cells (63.3%). Genes expressed in less than three cells were removed from the analyses. Expression data were log-normalized with the NormalizeData function, 2,000 highly variable genes were identified with FindVariableFeatures and scaling was performed with ScaleData regressing out the cell cycle scores, which were calculated with CellCycleScoring considering the human gene sets provided by Seurat.

After obtaining principal components with RunPCA, to characterize the cell populations present, we performed an unsupervised clustering analysis using the Louvain algorithm with a resolution of 0.8 in a shared nearest neighbors graph constructed with the first 20 principal components, as implemented in the FindClusters and FindNeighbors Seurat functions. Nonlinear dimensional reduction for visualization was done using the RunUMAP function with the same principal components. Markers in each cluster were identified with the FindAllMarkers function in the log-normalized counts using the Wilcoxon rank sum test. Genes with *P*-value < 0.05 (adjusted by Bonferroni’s correction), a log2 fold-change > 0.5 and a 10% difference in proportion of cells expressing them (specificity) were retained. Clusters were annotated in accordance with those markers. The plots were generated using the DimPlot, FeaturePlot, and VlnPlot functions from Seurat, as well as the ggplot2 and pheatmap R libraries.

### WISH.

We designed species-specific digoxigenin-labeled antisense riboprobes probes (*SI Appendix*, Table S5). Embryos at different developmental stages were fixed in 4% paraformaldehyde and dehydrated in methanol. WISH were performed as previously described (63). Embryos were imaged using the VHX-6000 (Keyence).

## Supplementary Material

Appendix 01 (PDF)

Dataset S01 (XLSX)

## Data Availability

DNA sequences' data have been deposited in NCBI and GEO (PRJNA983248 (27) and GSE264342 (44)). All other data are included in the manuscript and/or supporting information.
