# Supplementary material for: The dynamic behavior of chromatophores marks the transition from bands to spots in leopard geckos
Source: Proc Natl Acad Sci U S A. 2024 Jul 8;121(29):e2400486121. doi: 10.1073/pnas.2400486121 (PMC11260152; doi:10.1073/pnas.2400486121)
Supplement: Supplementary file 1 — Appendix 01 (PDF) [file pnas.2400486121.sapp.pdf]

Supporting Information for
The dynamic behavior of chromatophores marks the transition from bands to spots in leopard geckos

Asier Ullate-Agote[a,1] and Athanasia C. Tzika[a,2]

[a] Laboratory of Artificial & Natural Evolution (LANE), Department of Genetics & Evolution, University of Geneva, 1211 Geneva, Switzerland

[1] current address: Biomedical Engineering Program, Center for Applied Medical Research (CIMA), Universidad de Navarra, Instituto de Investigación Sanitaria de Navarra (IdiSNA), 31008 Pamplona, Spain

[2] To whom correspondence may be addressed. Email: athanasia.tzika@unige.ch

This PDF file includes:

Figures S1 to S17
Tables S1 to S5

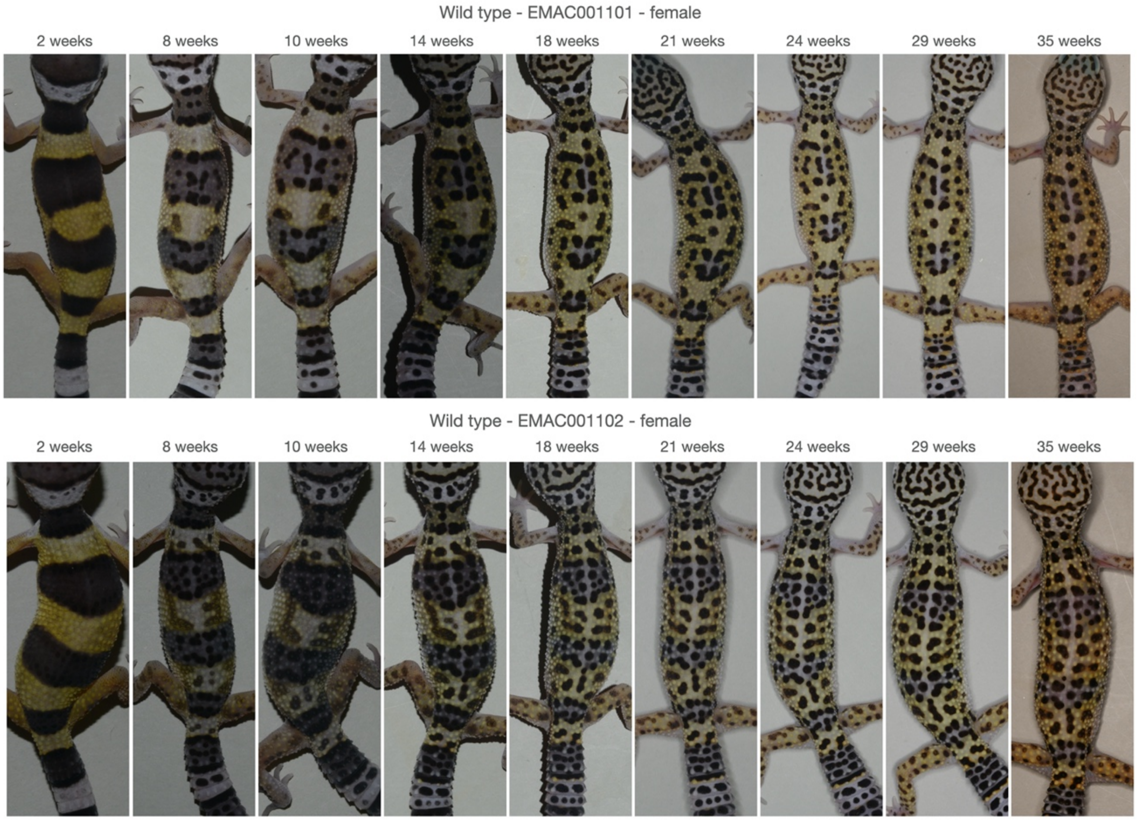

Figure S1. Ontogenetic changes of the wild type leopard gecko coloration pattern during the first 35 weeks after hatching.

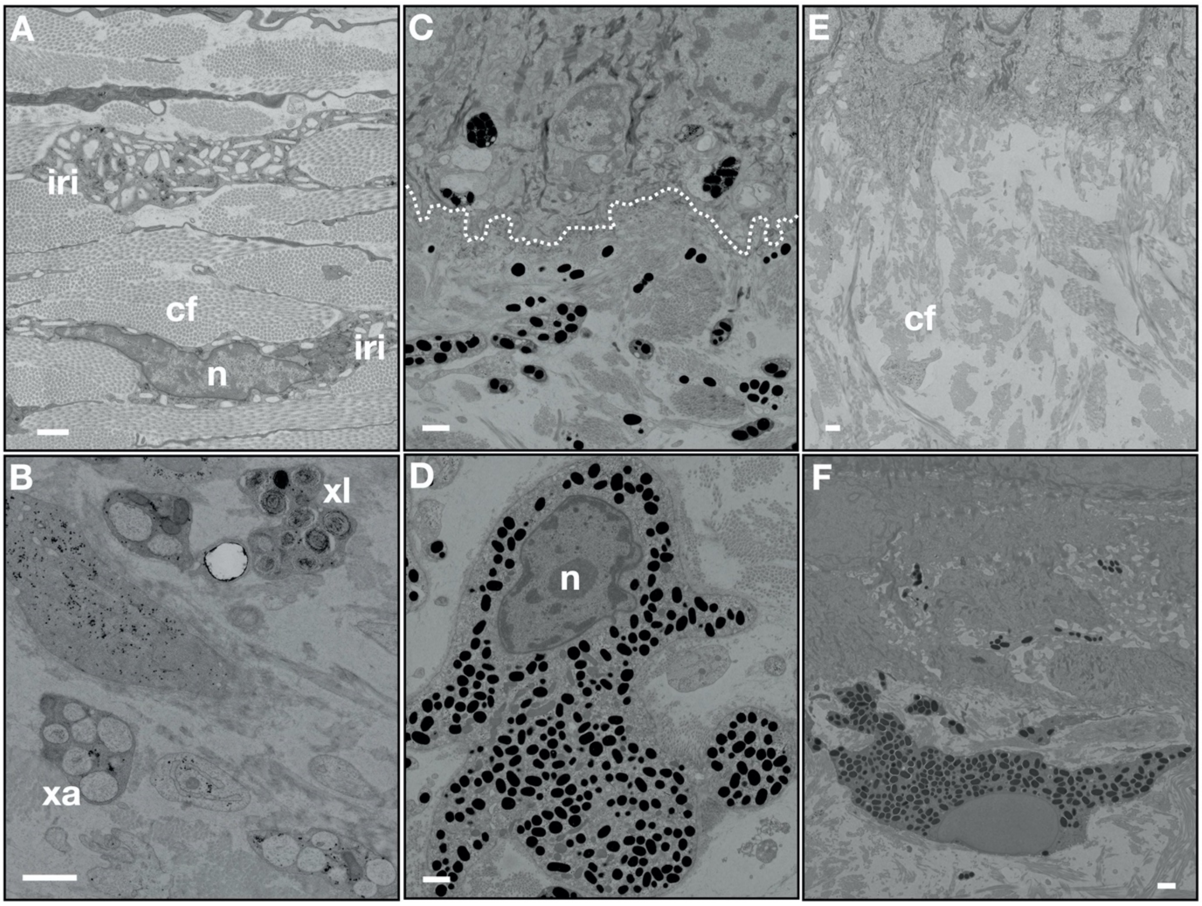

Figure S2. Transmission Electron Microscopy images of the leopard gecko skin. All images are from wild type animals unless otherwise specified. (A) Hatchling skin from the white head region. (B) Adult skin from the yellow region. (C) Adult skin from a dorsal black spot showing the presence of epidermal melanophores. The dotted line marks the epidermis-dermis boundary. (D) Dermal melanophore from the adult dorsal black spot. (E) Adult ventral skin where only collagen fibers are present. (F) Hatchling Mack Super Snow skin where only dermal and epidermal melanophores are present. iri: iridophores, cf: collagen fibers, n: nucleus, xa: xanthosomes with amorphous material possibly containing carotenoids, xl: xanthosomes with concentric lamellae possibly containing pteridines. Scale bars: 1 μm.

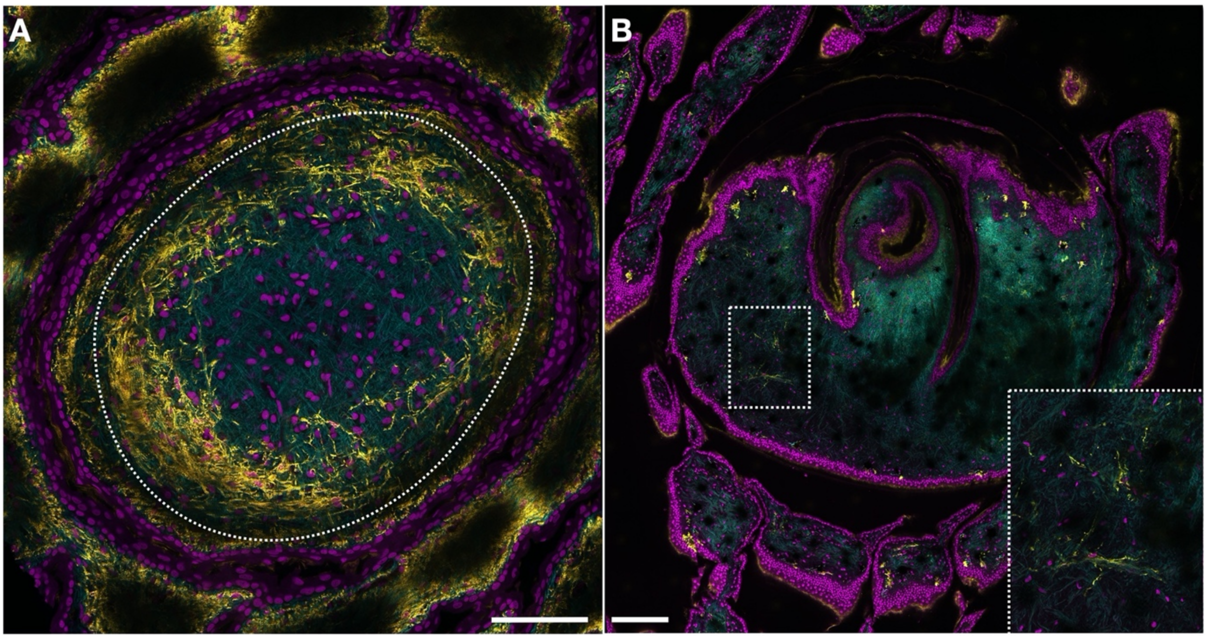

Figure S3. Fluorescent imaging of leopard gecko skin. (A) Transverse section of hatchling head white skin. Note in yellow the elongated shape of the iridophores within the dashed circle. (B) Transverse section of adult yellow skin. The section crosses the top of the scale. A magnification of the remaining iridophores is shown in the dotted inset. yellow: reflectance of the guanine crystals in the iridophores at 505 nm, green: collagen fibers, magenta: nuclei stained with TO-PRO-3. Scale bars: 100 µm (A), 200 µm (B).

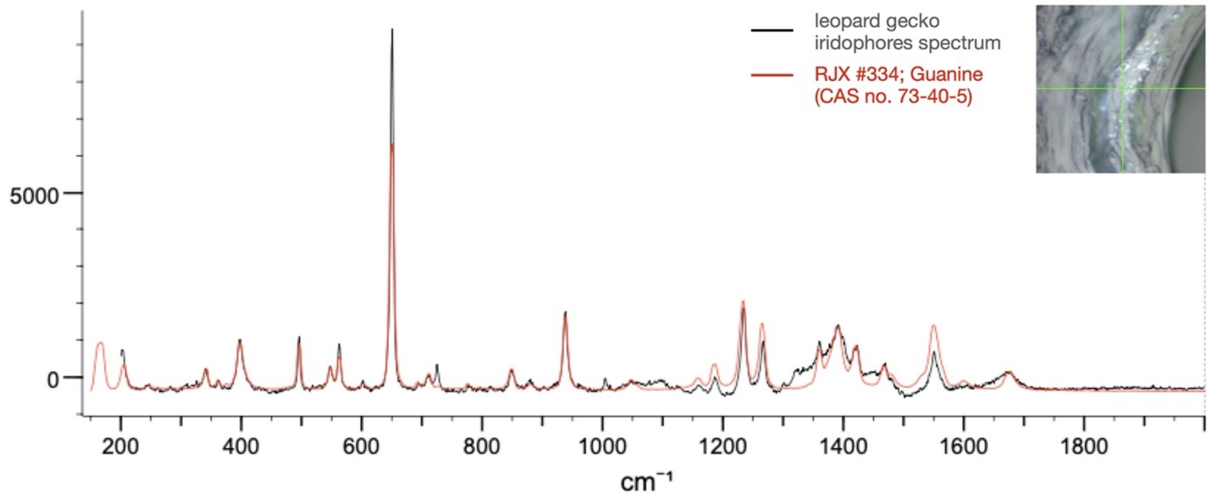

Figure S4. RAMAN microscopy of the leopard gecko iridophores. The spectrum was measured on a cryosection of the hatchling head white skin at the location indicated in the inset.

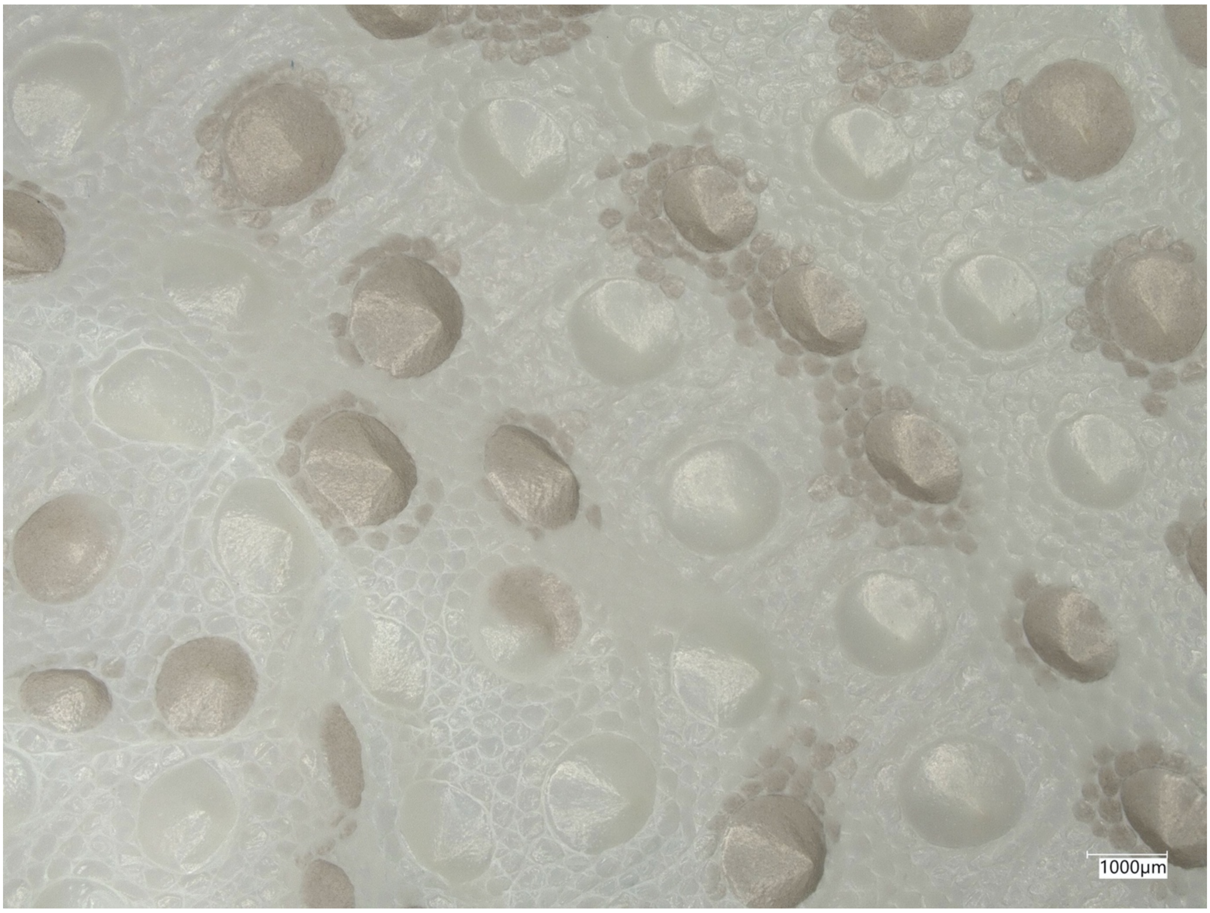

Figure S5. Shed of an adult leopard gecko. The black spots are visible on the shed, which is of epidermal origin.

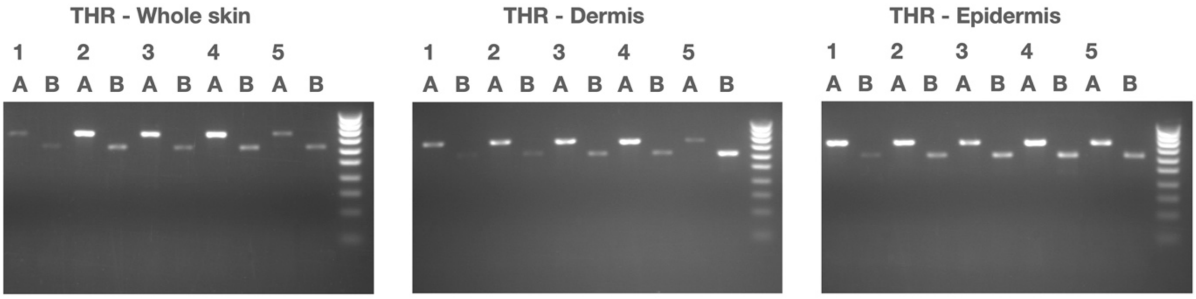

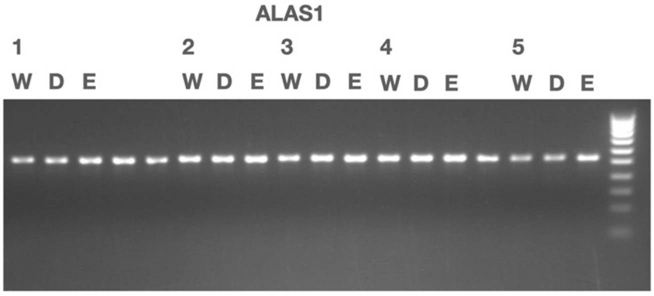

1	Adult	WT	yellow/black
2	Hatchling	WT	yellow
3	Hatchling	WT	black
4	Hatchling	WT	head white
5	Hatchling	MSS	dorsal black

Figure S6. Semi-quantitative amplification of thyroid hormone receptors A and B. The dermis and the epidermis were disassociated following on overnight Dispase I incubation at 4 ºC. The DNA ladder ranges from 100 to 1000 bp (SmartLadder SF, Eurogentec). W: whole skin, D: dermis, E: epidermis, A: *THRA*, B: *THRB*, WT: wild type, MSS: Mack Super Snow.

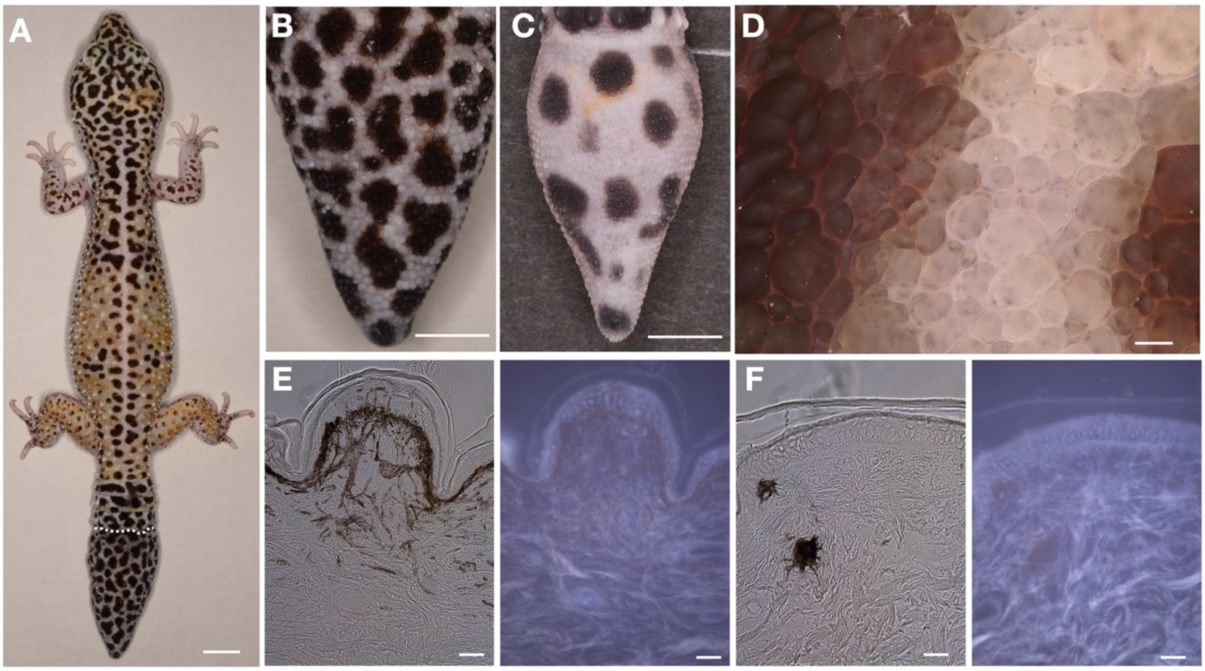

Figure S7. Histology of the regenerated tail. (A) Adult leopard gecko with a regenerated tail. The dashed line marks the boundary of the regenerated part. (B) Magnification of the regenerated tail of the animal in (A). (C) Magnification of the regenerated tail of a juvenile gecko with a banded dorsal pattern. A patch of xanthophores is also visible. (D) Greater magnification of (B) where the presence of melanophores is obvious among the black spots. (E) Transverse section of a black spot from the regenerated tail skin imaged with transmitted and coaxial reflected illumination. (F) Transverse section of a white part of the regenerated tail skin imaged with transmitted and coaxial reflected illumination. Iridophores are absent in both sections. Scale bars: 1 cm (A), 0.5 cm (B,C), 200 μm (D), 20 μm (E,F).

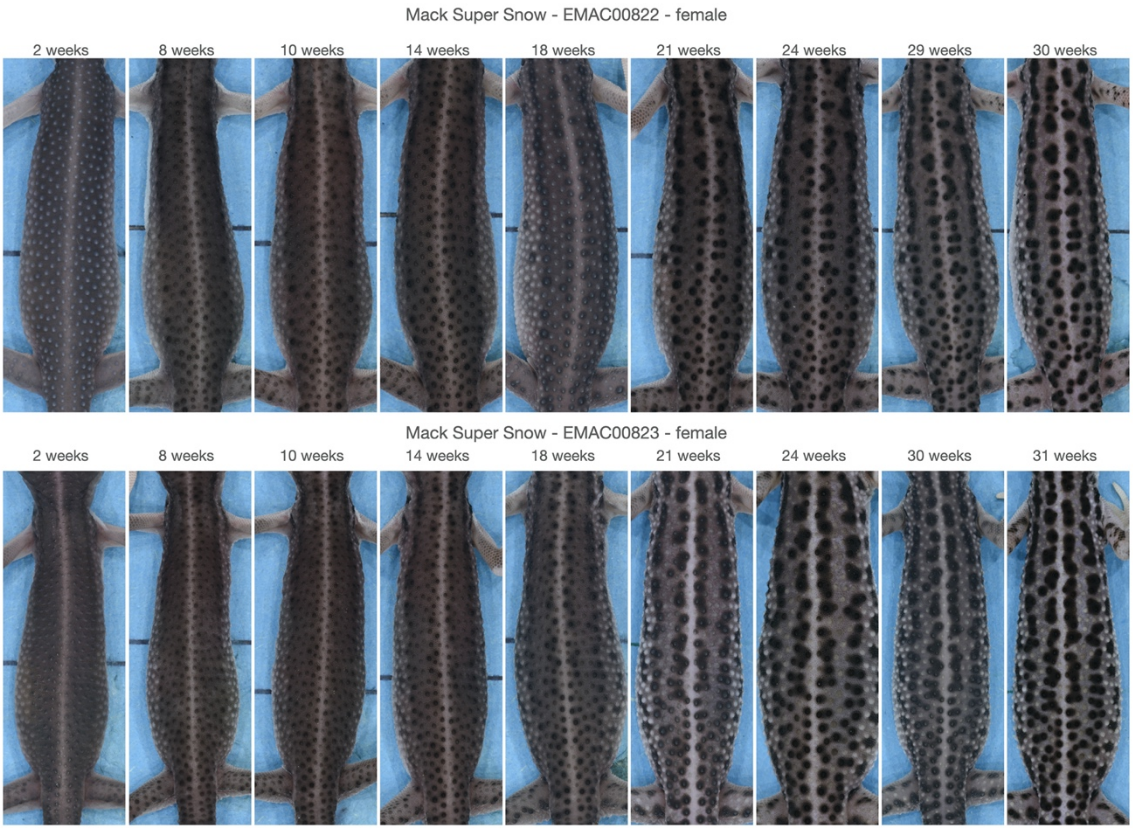

Figure S8. Ontogenetic changes of the Mack Super Snow leopard gecko coloration pattern during the first 30 and 31 weeks after hatching.

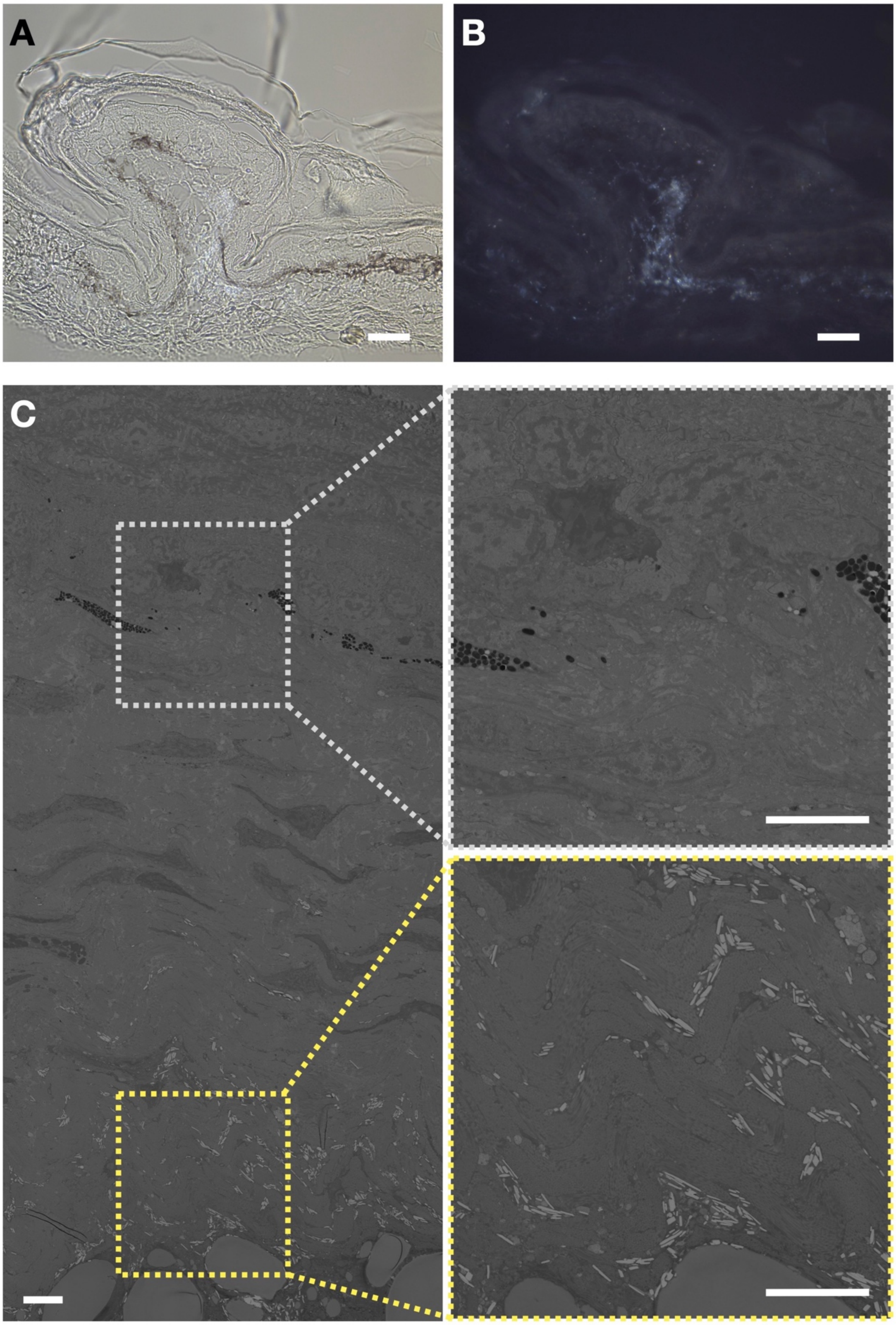

Figure S9. Iridophores are present in the white bands of the Mack Snow (*mss/+*) hatchlings as evidenced in xylene-treated paraffin sections with transmitted (A) and coaxial reflected illumination (B) and transmission electron microscopy (C). In the grey magnification window, we can appreciate the absence of xanthophores near the epidermis. In the yellow magnification window, the iridophores are visible deep in the dermis. Scale bars: 20 µm (A, B), 5 µm (C).

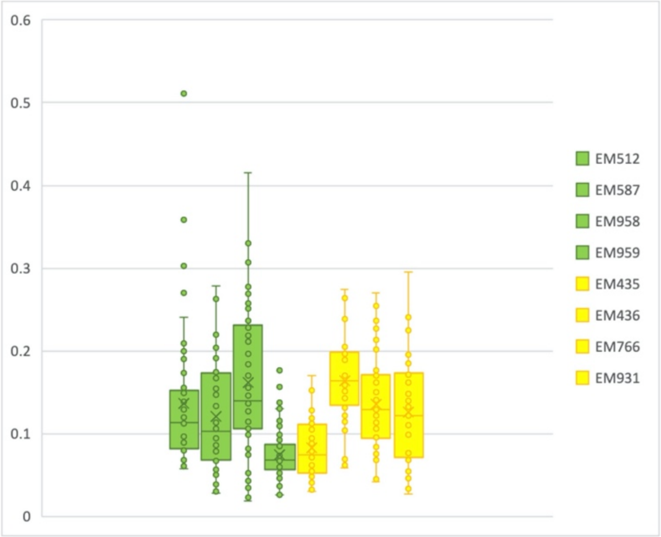

Figure S10. Area of black spots in wild-type and Mack Super Snow leopard geckos. Green box plots correspond to wild type animals, and yellow ones to Mack Super Snow. The area was measured in mm^2.

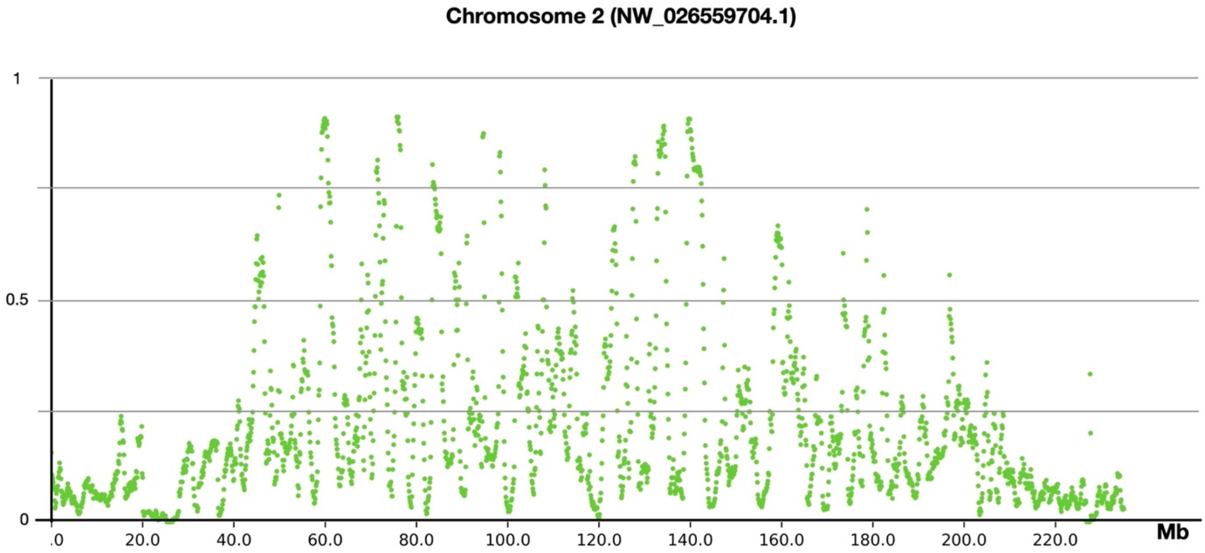

Figure S11. Co-segregation on Chromosome 2. Proportion (y-axis) of quality-filtered SNP/MNPs co-segregating with the Mack Super Snow locus on Chromosome 2. The proportion was calculated in overlapping windows of 1 Mb with a step of 100 Kb.

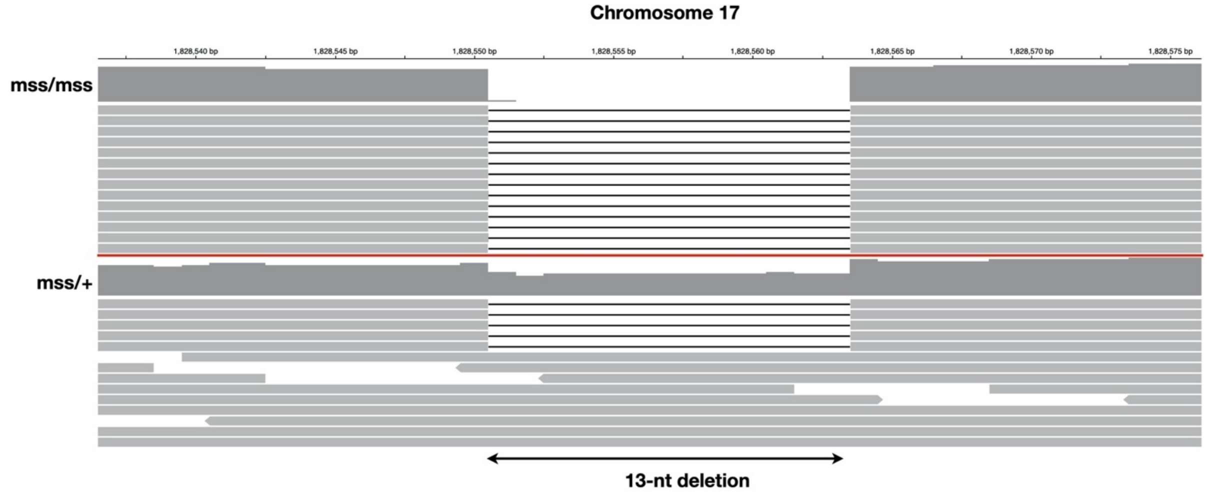

Figure S12. Thirteen nucleotide deletion in Chromosome 17 of Mack Super Snow. Alignment of the homozygous (*mss/mss*) and the heterozygous (*mss/+*) parental libraries to Chromosome 17. In light grey, the aligned reads and in dark grey, the coverage at this position of the Chromosome.

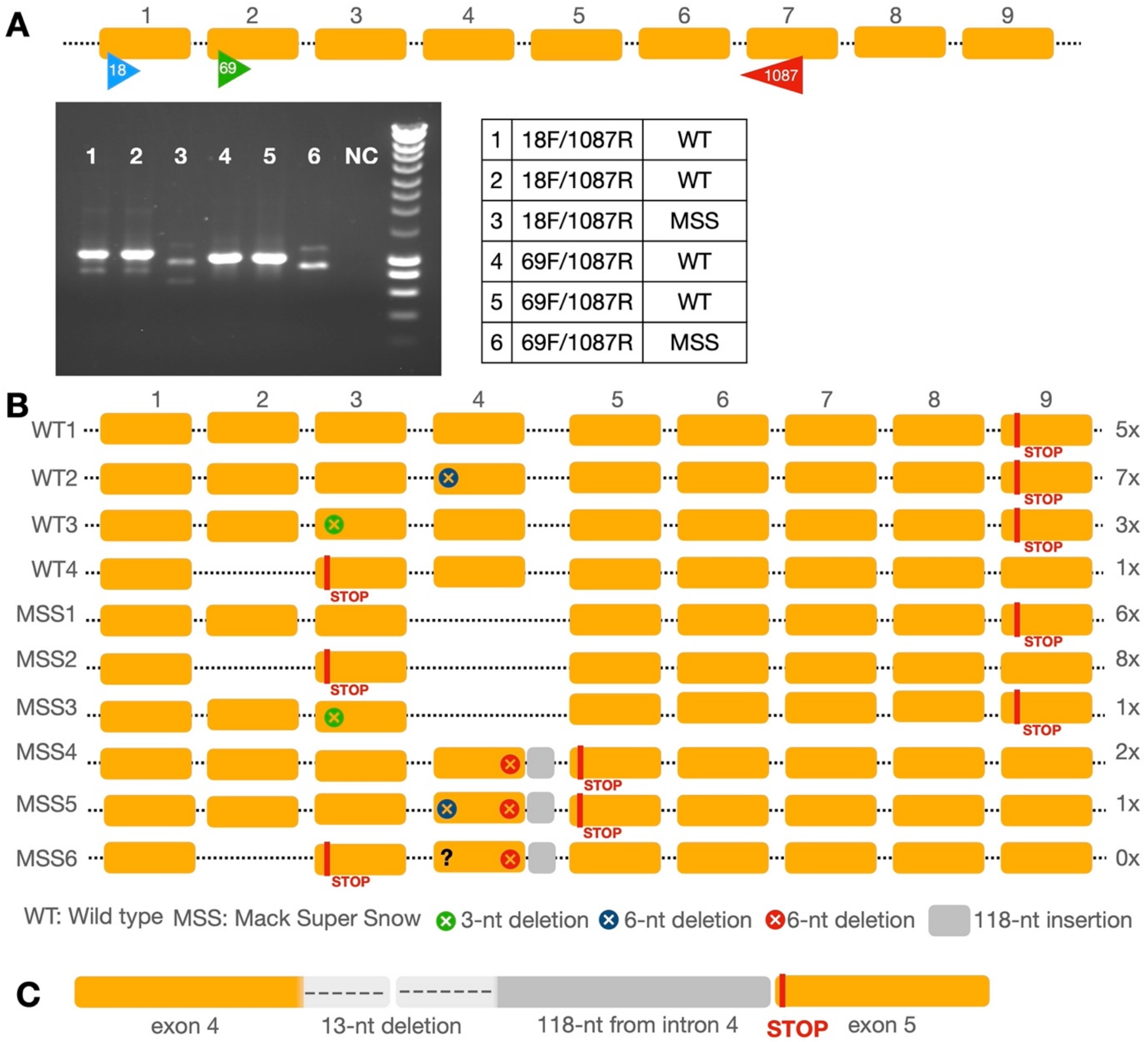

Figure S13. Leopard gecko *PAX7* isoforms. (A) Position of the primers used for the amplification of the *PAX7* isoforms in wild type and Mack Super Snow samples. (B) *PAX7* isoforms sequenced after cloning. The number on the right indicates the number of clones. Only MSS6 was not found possibly due to its low abundance. (C) Detail of the 118-nucleotide insertion in MSS4/5/6, which corresponds to the beginning of intron 4. The length of the exons is not in scale.

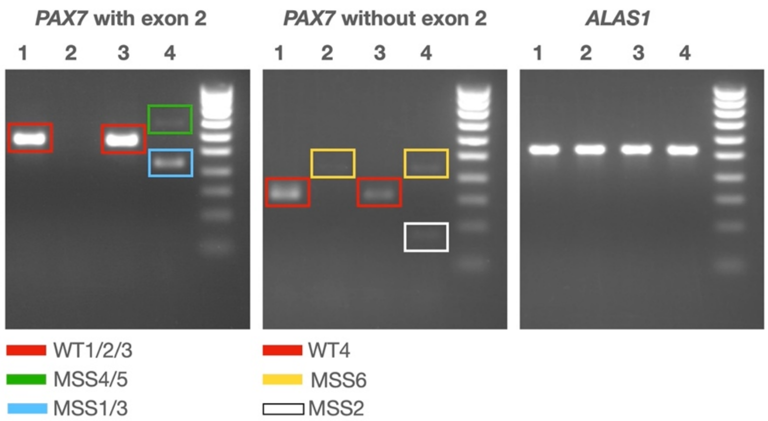

1	Dorsal skin S35	WT
2	Dorsal skin S35	MSS
3	Body part S35	WT
4	Body part S35	MSS

Figure S14. Semi-quantitative amplification of *PAX7* isoforms. The mRNA was extracted from embryos at the developmental stage 35. The DNA ladder ranges from 100 to 1000 bp (SmartLadder SF, Eurogentec).

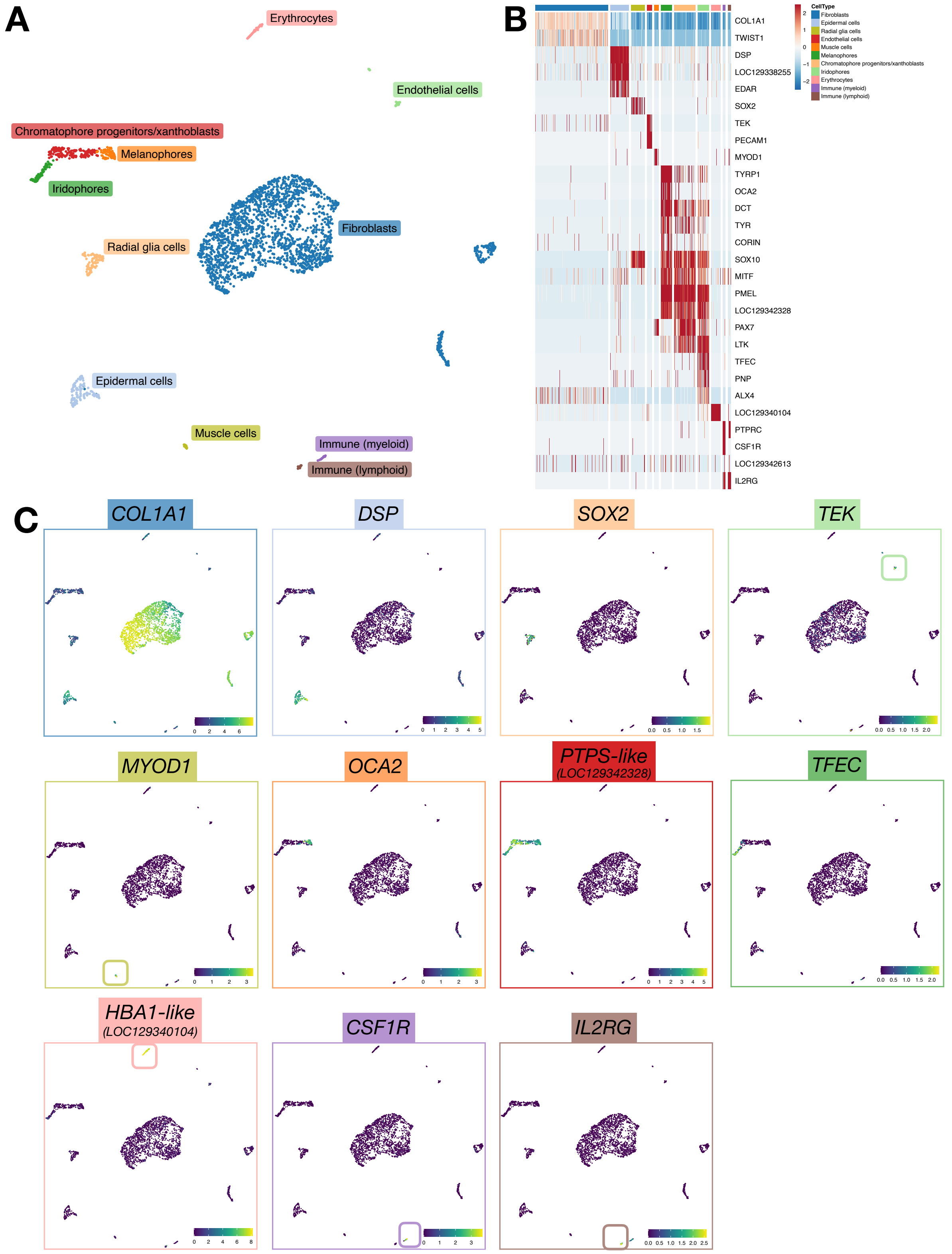

Figure S15. Single-cell transcriptomic data from the wild type embryonic dorsal skin. (A) UMAP with cell type assignment. (B) Heatmap with the expression level of selected markers for each cell type. (C) UMAP representations displaying the expression of the selected markers in all cells.

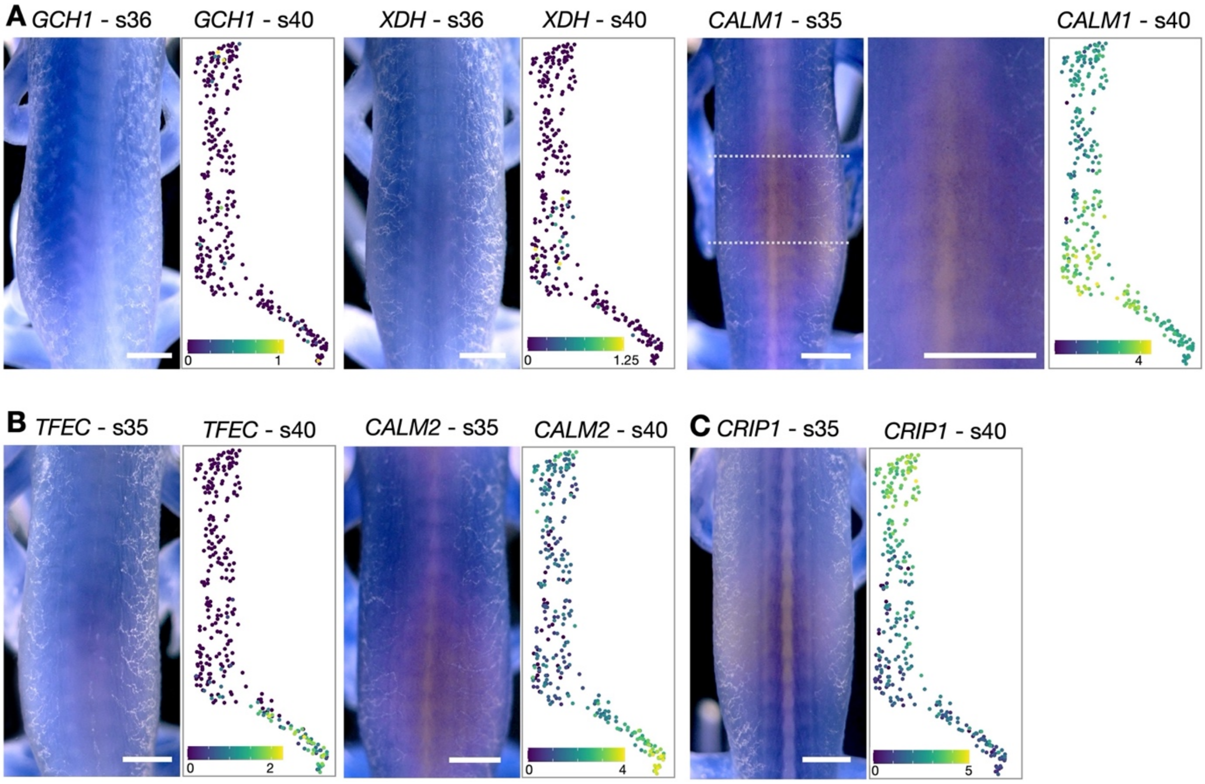

Figure S16. WISH and UMAP for classical and novel chromatophore markers. Dorsal view of stage 35 wild type embryos used for WISH with probes for classical and novel markers of (A) xanthophores, (B) iridophores, and (C) melanophores, and the accompanying UMAP representations, showing the expression of these genes in the chromatophores of stage 40 wild type embryos. Note the faint expression detected with the *CALM1* probe in a region that would correspond to a yellow band on the hatchling. This result should be taken with caution. With the *CRIP1* probe, the skeletal muscles are probably stained. Indeed, the single-cell transcriptomic analyses showed that, besides melanophores, this gene is also expressed in endothelial, muscle and immune (lymphoid) cells. Scale bars: 100 µm.

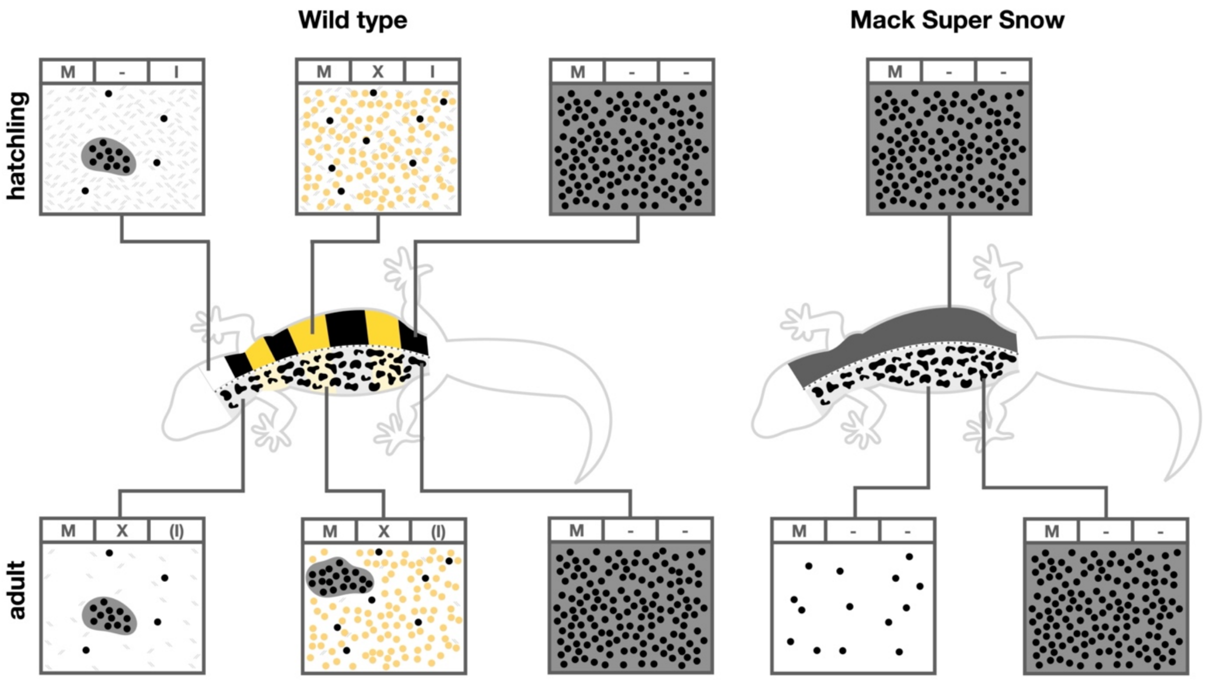

Figure S17. Graphical representation of the color pattern transition in wild type and Mack Super Snow leopard geckos. The number of iridophores is greatly reduced in the wild type adult geckos. Both xanthophores and iridophores are absent in Mack Super Snow geckos. Inspired by (52). M: melanophores, X: xanthophores, I: iridophores, (I): reduced number of iridophores.

Table S1. Genomic DNA libraries used for mapping the Mack Super Snow causative variant. In parentheses, we provide the total number of individuals in each library (column 'Source') and the average coverage for a 2.2 Gb genome (column 'Filtered reads'). PE: Paired-end reads.

Source	Genotype	Library type	Total reads	Filtered reads
Mack Super Snow male (1)	*mss/mss*	151 bp Illumina PE	199'699'441	183'686'372 (25.2x)
Mack Snow females (2)	*mss*/+	151 bp Illumina PE	189'344'321	174’016’385 (23.9x)
Mack Super Snow offspring (12)	*mss/mss*	151 bp Illumina PE	192'022'671	176'776'337 (24.3x)
Mack Snow offspring (14)	*mss*/+	151 bp Illumina PE	190'996'701	176'025'700 (24.2x)

Table S2. List of genes in the Mack Super Snow interval on Chromosome 17 based on the family mapping. We provide information on the presence of non synonymous modifications in the protein coding part of the genes detected in the Mack Super Snow libraries when compared to the wild type.

Start	End	Transcript ID	Transcript name	aa modifications
1726567	1730528	XM_055002085.1	KLHDC7A	NO
**1820721**	**1952340**	**XM_055001457.1**	**PAX7**	**6nt DEL**
1992752	2006839	XM_055001752.1	TAS1R2	YES
2008619	2033973	XM_055001702.1	ALDH4A1	YES
2036075	2089886	XM_055001358.1	IFFO2	NO
2114399	2226858	XM_055001525.1	UBR4	YES
2230853	2256802	XM_055001388.1	EMC1	NO
2257134	2262113	XM_055001473.1	MRTO4	NO
2262792	2269573	XM_055001324.1	LOC129344566 - AKR7L-like	NO
2270396	2280236	XM_055001325.1	SLC66A1	NO
2281593	2340374	XM_055001329.1	CAPZB	NO
2356282	2378776	XM_055001484.1	MICOS10	NO
2386646	2405687	XM_055001614.1	NBL1	NO
2410436	2432781	XM_055001584.1	HTR6	NO
2433073	2463626	XM_055001582.1	TMCO4	NO
2472845	2474123	XM_055001889.1	RNF186	YES
2475048	2491063	XM_055001753.1	TNFRSF8	YES
2505881	2515163	XM_055000982.1	MIIP	YES
2518347	2538484	XM_055001303.1	MFN2	NO
2539550	2560802	XM_055001965.1	PLOD1	NO
2562811	2569626	XM_055001478.1	KIAA2013	NO
2599300	2600959	XM_055001056.1	LOC129344432 - NPPC-like	NO
2620130	2622043	XM_055001754.1	LOC129344849 - NPPA-like	NO
**2632626**	**2655820**	**XM_055002056.1**	**CLCN6**	**6nt DEL**
2657821	2667692	XM_055001522.1	LOC129344689 - CLEC2D-like	YES
2668296	2688607	XM_055002006.1	MTHFR	YES
2690999	2714711	XM_055001755.1	C17H1orf167	YES
2716835	2728744	XM_055001996.1	AGTRAP	NO
2732570	2736750	XM_055002086.1	DRAXIN	NO
2747877	2758070	XM_055002107.1	MAD2L2	NO
2758127	2776955	XM_055001773.1	LOC129344863 - FBXO6-like	YES
2780427	2790060	XM_055000964.1	LOC129344374 - FBXO6-like	NO
2807166	2870506	XM_055001765.1	LOC129344858 - GOLIM4-like	NO
2879895	2880662	XM_055001757.1	FNDC10	NO
2903448	2904551	XM_055001410.1	LOC129344618 - ACTR2-like	NO
2906887	2954937	XM_055001723.1	SSU72	NO
2959851	2997408	XM_055001760.1	TMEM240	NO

Table S3. Genotyping for the 13-nucleotide deletion in *PAX7* of the Mack Super Snow family individuals used for the mapping.

ID	Phenotype	Genotype	13nt deletion
EM.A3.436	Mack Super Snow	mss/mss	-/-
EM.A3.500	Mack Snow	mss/+	+/-
EM.A3.510	Mack Snow	mss/+	+/-
EM.A3.436.2015.1	Mack Super Snow	mss/mss	-/-
EM.A3.436.2015.2	Mack Super Snow	mss/mss	-/-
EM.A3.436.2015.3	Mack Super Snow	mss/mss	-/-
EM.A3.436.2015.4	Mack Super Snow	mss/mss	-/-
EM.A3.436.2015.5	Mack Super Snow	mss/mss	-/-
EM.A3.436.2016.6	Mack Super Snow	mss/mss	-/-
EM.A3.436.2016.7	Mack Super Snow	mss/mss	-/-
EM.A3.436.2016.9	Mack Super Snow	mss/mss	-/-
EM.A3.436.2016.12	Mack Super Snow	mss/mss	-/-
EM.A3.436.2016.13	Mack Super Snow	mss/mss	-/-
EM.A3.436.2016.14	Mack Super Snow	mss/mss	-/-
EM.A3.436.2016.18	Mack Super Snow	mss/mss	-/-
EM.A3.436.2015.6	Mack Snow	mss/+	+/-
EM.A3.436.2015.7	Mack Snow	mss/+	+/-
EM.A3.436.2015.8	Mack Snow	mss/+	+/-
EM.A3.436.2016.1	Mack Snow	mss/+	+/-
EM.A3.436.2016.2	Mack Snow	mss/+	+/-
EM.A3.436.2016.3	Mack Snow	mss/+	+/-
EM.A3.436.2016.4	Mack Snow	mss/+	+/-
EM.A3.436.2016.5	Mack Snow	mss/+	+/-
EM.A3.436.2016.8	Mack Snow	mss/+	+/-
EM.A3.436.2016.10	Mack Snow	mss/+	+/-
EM.A3.436.2016.11	Mack Snow	mss/+	+/-
EM.A3.436.2016.15	Mack Snow	mss/+	+/-
EM.A3.436.2016.16	Mack Snow	mss/+	+/-
EM.A3.436.2016.17	Mack Snow	mss/+	+/-

Table S4. Genotyping for the 13-nucleotide deletion in *PAX7* of 190 individuals in our colony originating from 24 unrelated lineages obtained to study different morphs and from different breeders. Individuals found homozygous for the deletion are marked' as '-/-', heterozygous as '+/-' and those lacking the deletion as '+/+'.

ID	Phenotype	Genotype	13nt deletion	Lineage
EM.A4.435.2016.1	Mack Snow	mss/+	+/-	11, 12
EM.A4.435.2016.2	Mack Snow	mss/+	+/-	11, 12
EM.A4.435.2016.3	Mack Snow	mss/+	+/-	11, 12
EM.A4.435.2016.4	Mack Super Snow	mss/mss	-/-	11, 12
EM.A4.435.2016.5	Mack Snow	mss/+	+/-	11, 12
EM.A4.435.2016.6	Mack Snow	mss/+	+/-	11, 12
EM.A5.313.2016.1	Mack Snow	mss/+	+/-	8, 11
EM.A5.313.2016.2	Mack Super Snow	mss/mss	-/-	8, 11
EM.A5.313.2016.3	Mack Super Snow	mss/mss	-/-	8, 11
EM.A5.313.2016.4	Mack Super Snow	mss/mss	-/-	8, 11
EM.A5.313.2016.5	Mack Snow	mss/+	+/-	8, 11
EM.A5.313.2016.6	Mack Super Snow	mss/mss	-/-	8, 11
EM.A5.313.2016.7	Mack Snow	mss/+	+/-	8, 11
EM.A6.550.2016.1	Mack Snow	mss/+	+/-	9, 12
EM.A6.550.2016.2	Mack Snow	mss/+	+/-	9, 12
EM.A6.550.2016.5	Mack Snow	mss/+	+/-	9, 12
EM.A7.520.2016.1	Mack Snow	mss/+	+/-	9, 11, 12
EM.A7.520.2016.2	Mack Snow	mss/+	+/-	9, 11, 12
EM.A7.520.2016.3	Mack Super Snow	mss/mss	-/-	9, 11, 12
EM.A7.520.2016.4	Mack Super Snow	mss/mss	-/-	9, 11, 12
EM.A8.588.2016.1	Mack Snow	mss/+	+/-	2, 11, 12
EM.A8.588.2016.10	Mack Snow	mss/+	+/-	2, 11, 12
EM.A8.588.2016.11	Mack Snow	mss/+	+/-	2, 11, 12
EM.A8.588.2016.12	Mack Snow	mss/+	+/-	2, 11, 12
EM.A8.588.2016.13	Mack Snow	mss/+	+/-	2, 11, 12
EM.A8.588.2016.2	Mack Snow	mss/+	+/-	2, 11, 12
EM.A8.588.2016.3	Mack Super Snow	mss/mss	-/-	2, 11, 12
EM.A8.588.2016.4	Mack Snow	mss/+	+/-	2, 11, 12
EM.A8.588.2016.5	Mack Super Snow	mss/mss	-/-	2, 11, 12
EM.A8.588.2016.6	Mack Snow	mss/+	+/-	2, 11, 12
EM.A8.588.2016.7	Mack Snow	mss/+	+/-	2, 11, 12
EM.A8.588.2016.8	Mack Snow	mss/+	+/-	2, 11, 12
EM.A8.588.2016.9	Mack Snow	mss/+	+/-	2, 11, 12
EM.B1.344.2016.1	Mack Snow	mss/+	+/-	11, 12, 16
EM.B3.542.2016.1	Mack Snow	mss/+	+/-	2, 7, 9, 11, 12
EM.B3.542.2016.2	Mack Snow	mss/+	+/-	2, 7, 9, 11, 12
EM.B3.542.2016.3	Mack Super Snow	mss/mss	-/-	2, 7, 9, 11, 12
EM.B3.542.2016.4	Mack Super Snow	mss/mss	-/-	2, 7, 9, 11, 12
EM.B3.542.2016.5	Mack Snow	mss/+	+/-	2, 7, 9, 11, 12
EM.B3.542.2016.6	Mack Super Snow	mss/mss	-/-	2, 7, 9, 11, 12
EM.B3.542.2016.7	Mack Snow	mss/+	+/-	2, 7, 9, 11, 12
EM.C1.502.2016.1	Mack Snow	mss/+	+/-	9, 11, 12
EM.C1.502.2016.2	other	+/+	+/+	9, 11, 12
EM.C1.502.2016.3	Mack Snow	mss/+	+/-	9, 11, 12
EM.C1.502.2016.4	Mack Snow	mss/+	+/-	9, 11, 12
EM.C1.502.2016.10	Mack Snow	mss/+	+/-	9, 11, 12
EM.C1.502.2016.5	other	+/+	+/+	9, 11, 12
EM.C1.502.2016.6	other	+/+	+/+	9, 11, 12
EM.C1.502.2016.7	Mack Snow	mss/+	+/-	9, 11, 12
EM.C1.502.2016.8	other	+/+	+/+	9, 11, 12
EM.C1.502.2016.9	Mack Snow	mss/+	+/-	9, 11, 12
EM.C2.504.2016.1	Mack Snow	mss/+	+/-	3, 4, 6, 9, 11, 12
EM.C2.504.2016.2	other	+/+	+/+	3, 4, 6, 9, 11, 12
EM.C3.515.2016.1	Mack Snow	mss/+	+/-	1, 5, 10, 11, 12

EM.C3.515.2016.10	Mack Snow	mss/+	+/-	1, 5, 10, 11, 12
EM.C3.515.2016.11	Mack Snow	mss/+	+/-	1, 5, 10, 11, 12
EM.C3.515.2016.12	Mack Snow	mss/+	+/-	1, 5, 10, 11, 12
EM.C3.515.2016.13	Mack Snow	mss/+	+/-	1, 5, 10, 11, 12
EM.C3.515.2016.2	other	+/+	+/+	1, 5, 10, 11, 12
EM.C3.515.2016.3	other	+/+	+/+	1, 5, 10, 11, 12
EM.C3.515.2016.4	other	+/+	+/+	1, 5, 10, 11, 12
EM.C3.515.2016.5	other	+/+	+/+	1, 5, 10, 11, 12
EM.C3.515.2016.6	Mack Super Snow	mss/mss	-/-	1, 5, 10, 11, 12
EM.C3.515.2016.7	Mack Super Snow	mss/mss	-/-	1, 5, 10, 11, 12
EM.C3.515.2016.8	Mack Snow	mss/+	+/-	1, 5, 10, 11, 12
EM.C3.515.2016.9	other	+/+	+/+	1, 5, 10, 11, 12
EM.J1.411.2017.10	other	+/+	+/+	18, 19, 20
EM.J1.411.2017.11	other	+/+	+/+	18, 19, 20
EM.J1.411.2017.12	other	+/+	+/+	18, 19, 20
EM.J1.411.2017.15	other	+/+	+/+	18, 19, 20
EM.J1.411.2017.16	other	+/+	+/+	18, 19, 20
EM.J1.411.2017.25	other	+/+	+/+	18, 19, 20
EM.J1.411.2017.26	other	+/+	+/+	18, 19, 20
EM.J1.411.2017.29	other	+/+	+/+	18, 19, 20
EM.J1.411.2017.30	other	+/+	+/+	18, 19, 20
EM.J1.411.2017.31	other	+/+	+/+	18, 19, 20
EM.J1.411.2017.9	other	+/+	+/+	18, 19, 20
EM.J2.516.2016.1	Mack Snow	mss/+	+/-	1, 11, 18, 19, 20
EM.J2.516.2016.2	other	+/+	+/+	1, 11, 18, 19, 20
EM.J2.516.2016.3	Mack Snow	mss/+	+/-	1, 11, 18, 19, 20
EM.K2.579.2016.1	other	+/+	+/+	1, 18, 19, 20
EM.K2.579.2016.2	other	+/+	+/+	1, 18, 19, 20
EM.K2.579.2016.3	Mack Snow	mss/+	+/-	1, 18, 19, 20
EM.K2.579.2016.4	other	+/+	+/+	1, 18, 19, 20
EM.K2.579.2017.1	other	+/+	+/+	1, 18, 19, 20
EM.K2.579.2017.10	other	+/+	+/+	1, 18, 19, 20
EM.K2.579.2017.11	other	+/+	+/+	1, 18, 19, 20
EM.K2.579.2017.17	other	+/+	+/+	1, 18, 19, 20
EM.K2.579.2017.18	other	+/+	+/+	1, 18, 19, 20
EM.K2.579.2017.19	other	+/+	+/+	1, 18, 19, 20
EM.K2.579.2017.2	other	+/+	+/+	1, 18, 19, 20
EM.K2.579.2017.20	other	+/+	+/+	1, 18, 19, 20
EM.K2.579.2017.21	Mack Snow	mss/+	+/-	1, 18, 19, 20
EM.K2.579.2017.22	other	+/+	+/+	1, 18, 19, 20
EM.K2.579.2017.23	other	+/+	+/+	1, 18, 19, 20
EM.K2.579.2017.3	other	+/+	+/+	1, 18, 19, 20
EM.K2.579.2017.4	other	+/+	+/+	1, 18, 19, 20
EM.K2.579.2017.5	other	+/+	+/+	1, 18, 19, 20
EM.K2.579.2017.6	other	+/+	+/+	1, 18, 19, 20
EM.K2.579.2017.7	other	+/+	+/+	1, 18, 19, 20
EM.K2.579.2017.8	other	+/+	+/+	1, 18, 19, 20
EM.K2.579.2017.9	Mack Snow	mss/+	+/-	1, 18, 19, 20
EM.K3.392.2016.1	other	+/+	+/+	18, 19, 20
EM.K3.392.2016.2	other	+/+	+/+	18, 19, 20
EM.K3.392.2016.3	other	+/+	+/+	18, 19, 20
EM.K3.392.2016.7	other	+/+	+/+	18, 19, 20
EM.K3.392.2017.1	other	+/+	+/+	18, 19, 20
EM.K3.392.2017.10	other	+/+	+/+	18, 19, 20
EM.K3.392.2017.12	other	+/+	+/+	18, 19, 20
EM.K3.392.2017.13	other	+/+	+/+	18, 19, 20
EM.K3.392.2017.14	other	+/+	+/+	18, 19, 20
EM.K3.392.2017.17	other	+/+	+/+	18, 19, 20
EM.K3.392.2017.18	other	+/+	+/+	18, 19, 20
EM.K3.392.2017.2	other	+/+	+/+	18, 19, 20

EM.K3.392.2017.3	other	+/+	+/+	18, 19, 20
EM.K3.392.2017.8	other	+/+	+/+	18, 19, 20
EM.K3.392.2017.21	other	+/+	+/+	18, 19, 20
EM.K3.392.2017.22	other	+/+	+/+	18, 19, 20
EM.K3.392.2017.25	other	+/+	+/+	18, 19, 20
EM.K3.392.2017.26	other	+/+	+/+	18, 19, 20
EM.K3.392.2017.28	other	+/+	+/+	18, 19, 20
EM.K3.392.2017.29	other	+/+	+/+	18, 19, 20
EM.K3.392.2017.30	other	+/+	+/+	18, 19, 20
EM.K3.392.2017.31	other	+/+	+/+	18, 19, 20
EM.K3.392.2017.33	other	+/+	+/+	18, 19, 20
EM.K3.392.2017.4	other	+/+	+/+	18, 19, 20
EM.K3.392.2017.5	other	+/+	+/+	18, 19, 20
EM.K3.392.2017.6	other	+/+	+/+	18, 19, 20
EM.K3.392.2017.9	other	+/+	+/+	18, 19, 20
EM.L2.540.2017.1	Mack Snow	mss/+	+/-	18, 19, 22
EM.L2.540.2017.10	other	+/+	+/+	18, 19, 22
EM.L2.540.2017.11	Mack Snow	mss/+	+/-	18, 19, 22
EM.L2.540.2017.2	other	+/+	+/+	18, 19, 22
EM.L2.540.2017.3	other	+/+	+/+	18, 19, 22
EM.L2.540.2017.4	Mack Snow	mss/+	+/-	18, 19, 22
EM.L2.540.2017.5	Mack Snow	mss/+	+/-	18, 19, 22
EM.L2.540.2017.8	other	+/+	+/+	18, 19, 22
EM.L2.540.2017.9	other	+/+	+/+	18, 19, 22
EM.L3.529.2017.1	other	+/+	+/+	18, 21, 22, 23
EM.L3.529.2017.10	other	+/+	+/+	18, 21, 22, 23
EM.L3.529.2017.17	other	+/+	+/+	18, 21, 22, 23
EM.L3.529.2017.18	other	+/+	+/+	18, 21, 22, 23
EM.L3.529.2017.19	other	+/+	+/+	18, 21, 22, 23
EM.L3.529.2017.2	other	+/+	+/+	18, 21, 22, 23
EM.L3.529.2017.3	other	+/+	+/+	18, 21, 22, 23
EM.L3.529.2017.4	other	+/+	+/+	18, 21, 22, 23
EM.L3.529.2017.5	other	+/+	+/+	18, 21, 22, 23
EM.L3.529.2017.6	other	+/+	+/+	18, 21, 22, 23
EM.L3.529.2017.7	other	+/+	+/+	18, 21, 22, 23
EM.L3.529.2017.8	other	+/+	+/+	18, 21, 22, 23
EM.L3.529.2017.9	other	+/+	+/+	18, 21, 22, 23
EM.O2.518.2017.19	other	+/+	+/+	24
EM.O2.518.2017.3	Mack Snow	mss/+	+/-	24
EM.O2.518.2017.5	other	+/+	+/+	24
EM.O2.518.2017.6	other	+/+	+/+	24
EM.O2.518.2017.7	Mack Snow	mss/+	+/-	24
EM.O2.518.2017.9	other	+/+	+/+	24
EM.S2.457.2017.1	other	+/+	+/+	13, 14, 15
EM.S2.457.2017.7	other	+/+	+/+	13, 14, 15
EM.T1.293.2017.4	other	+/+	+/+	14, 15, 16, 17
EM.T1.293.2017. 5	other	+/+	+/+	14, 15, 16, 17

Table S5. Primers used in this study.

Variant calling on gRNA	Forward	Pax7_F4	GAGTTCAATCAGCCGTGTGC
Variant calling on gRNA	Reverse	Pax7_R3	CCAGGCCCTGTTCTCGATTT
Isoform amplification on cDNA	Forward	Pax7_18B-F	GGACCGTCCCGAGGATGATG
Isoform amplification on cDNA	Forward	Pax7_69-F	CCGGATTCCCTCTCGAAGTC
Isoform amplification on cDNA	Forward	Pax7_72-F	ATTCCCTCTCGAAGCAGGTC
Isoform amplification on cDNA	Reverse	Pax7_625-R	TTCAGACTCGACGTCAGAGC
Isoform amplification on cDNA	Reverse	Pax7_1087-R	GGTGTCCGAGTAGCTGGAAA
THRA amplification on cDNA	Forward	THRA_1065-F	GTCAGTCACCAATGGCCTCC
THRA amplification on cDNA	Reverse	THRA_1798-R	GTGGCCCAAGTCCAGACAT
THRB amplification on cDNA	Forward	THRB_819-F	GTTGGATGACAGCAAGCGAT
THRB amplification on cDNA	Reverse	THRB_1463-R	CCTGGGCGATCTGAAGACAT
ALAS amplification on cDNA	Forward	ALAS1_qF1	TCTACCACTGAGAAGGCCAA
ALAS amplification on cDNA	Reverse	ALAS1_713-R	TTGCTGGTCTGGGAATCTGA
In situ probe amplification on cDNA	Forward	PMEL-73-F	GACTGTACCCCAGGTGGAAG
In situ probe amplification on cDNA	Reverse	PMEL-720-R	AGTAGGAGATGTCAGCGTCG
In situ probe amplification on cDNA	Forward	Pax7-722-F	ACCCGGATATCTACACCCGA
In situ probe amplification on cDNA	Reverse	Pax7-1487-R	GGTAGGAGGCCTAACACAGC
In situ probe amplification on cDNA	Forward	GCH1c_166F	GCTCTGGAGGCTGCCTAC
In situ probe amplification on cDNA	Reverse	GCH1c_618R	TTTCTGAACGCCCCTCATGA
In situ probe amplification on cDNA	Forward	XDHc_526F	AGTGGGAGCAAGCTGACTTT
In situ probe amplification on cDNA	Reverse	XDHc_1275R	ATGGGCTTTGGTGCTGGTAA
In situ probe amplification on cDNA	Forward	CALM1c_238F	TGGCACCATCACAACAAAAGA
In situ probe amplification on cDNA	Reverse	CALM1c_781R	GGAACAGGACCACCAACCAT
In situ probe amplification on cDNA	Forward	TFECc_188F	CAACAGCGACCAAGGAATGC
In situ probe amplification on cDNA	Reverse	TFECc_900R	AGCTACTTCTTCGGCTGCTC
In situ probe amplification on cDNA	Forward	CALM2c_174F	ACTGACAGAGGAACAGATTGCA
In situ probe amplification on cDNA	Reverse	CALM2c_828R	TCCAGAGTAAGCCACATGCA
In situ probe amplification on cDNA	Forward	CRIP1c_103F	CCATGCCCAAGTGTCCCC
In situ probe amplification on cDNA	Reverse	CRIP1c_521R	TGCTTATCCACCCTGTCATGT

Sequences of the leopard gecko wild type *PAX7* isoforms. The protein translation is provided. The starting site is highlighted in green, while the stop codon is in red.

>PAX7_isoformWT1

```
atggctgcgctgcccgggaccgtcccgaggatgatgcgcccggcgcccggccagaactac
 M  A  A  L  P  G  T  V  P  R  M  M  R  P  A  P  G  Q  N  Y
ccgcgcaccggattccctctcgaagtctccacgcccctgggccaaggccgggtcaaccag
 P  R  T  G  F  P  L  E  V  S  T  P  L  G  Q  G  R  V  N  Q
ctcgggggggtcttcatcaacgggcggcccctgcccaaccacatccgccacaagatcgtg
 L  G  G  V  F  I  N  G  R  P  L  P  N  H  I  R  H  K  I  V
gagatggcccaccacggcatccggccctgcgtcatctcccggcagctgcgcgtctcccac
 E  M  A  H  H  G  I  R  P  C  V  I  S  R  Q  L  R  V  S  H
gggtgcgtctccaagatcctttgccgctaccaggagacgggctcgatccgccccggggcc
 G  C  V  S  K  I  L  C  R  Y  Q  E  T  G  S  I  R  P  G  A
atcggaggcagcaagcccaggcaggtcgcgactcccgacgtggagaaaaaaattgaagaa
 I  G  G  S  K  P  R  Q  V  A  T  P  D  V  E  K  K  I  E  E
tataagcgcgaaaatccaggcatgttcagctgggagatccgggaccggctcctcaaagac
 Y  K  R  E  N  P  G  M  F  S  W  E  I  R  D  R  L  L  K  D
ggccactgcgaccggagtacagtcccttcaggtttagtgagttcaatcagccgtgtgctc
 G  H  C  D  R  S  T  V  P  S  G  L  V  S  S  I  S  R  V  L
cgaatcaagttcgggaagaaggaggaggaggaagattgcgacaagaaagaggaggatggc
 R  I  K  F  G  K  K  E  E  E  E  D  C  D  K  K  E  E  D  G
gagaagaaagccaagcacagcatcgacggcatcctgggagataaaggtaaccgactggat
 E  K  K  A  K  H  S  I  D  G  I  L  G  D  K  G  N  R  L  D
gaaggctctgacgtcgagtctgaaccggacctgcccctgaagcgcaagcagcgccgcagc
 E  G  S  D  V  E  S  E  P  D  L  P  L  K  R  K  Q  R  R  S
cggaccaccttcactgcggaacagttggaggagctggagaaggcctttgagaggacccat
 R  T  T  F  T  A  E  Q  L  E  E  L  E  K  A  F  E  R  T  H
tacccggatatctacacccgagaagagcttgctcagcggaccaaactcactgaggcccgg
 Y  P  D  I  Y  T  R  E  E  L  A  Q  R  T  K  L  T  E  A  R
gtccaggtctggttcagcaaccgaagagcacggtggcgtaagcaagcaggagccaaccag
 V  Q  V  W  F  S  N  R  R  A  R  W  R  K  Q  A  G  A  N  Q
ctagcagcttttaaccacctcttgccaggggcttcccacccactgggatgccagctttg
 L  A  A  F  N  H  L  L  P  G  G  F  P  P  T  G  M  P  A  L
ccaccctatcagcttccagattcgacctacccaacagccgccatttcccaagacgggggc
 P  P  Y  Q  L  P  D  S  T  Y  P  T  A  A  I  S  Q  D  G  G
agcaccgtgcacagaccccagcctctcccgccgtccaccatgcaccaggggggattggca
 S  T  V  H  R  P  Q  P  L  P  P  S  T  M  H  Q  G  G  L  A
gccgccgccgccgactccagctcagcgtatggggcccggcacagcttttccagctactcg
 A  A  A  A  D  S  S  S  A  Y  G  A  R  H  S  F  S  S  Y  S
gacaccttcatgaaccctgcagctcccacaaaccacatgaaccccgtcagcaacggcctc
 D  T  F  M  N  P  A  A  P  T  N  H  M  N  P  V  S  N  G  L
tcccctcaggtgatgagcatcctcagcaaccccagtggagtcccaccgcagccccagggc
 S  P  Q  V  M  S  I  L  S  N  P  S  G  V  P  P  Q  P  Q  G
gacttttccatctccccccctccacggcagcttggacactgccaattccatctcggccagc
 D  F  S  I  S  P  L  H  G  S  L  D  T  A  N  S  I  S  A  S
tgcagccagaggagcgactccatcaagtccgtcgacagcctccccacttcacaatcctac
 C  S  Q  R  S  D  S  I  K  S  V  D  S  L  P  T  S  Q  S  Y
tgtcccccgacctacaccaccactggctacagcgtggaccccgtggcaggctaccagtat
 C  P  P  T  Y  T  T  T  G  Y  S  V  D  P  V  A  G  Y  Q  Y
gggcaatatgggcaaactgcagttgactacctggccaaaaacgtgagcctgtctacacaa
 G  Q  Y  G  Q  T  A  V  D  Y  L  A  K  N  V  S  L  S  T  Q
cgcagaatgaagctcggggagcattccgctgtgttaggcctcctacccgtggaaacgggc
 R  R  M  K  L  G  E  H  S  A  V  L  G  L  L  P  V  E  T  G
caagcttactaatggagacacttgcagtctgctatcaaggagaagaaaaatccccctctg
 Q  A  Y  -  W  R  H  L  Q  S  A  I  K  E  K  K  N  P  P  L
ccaccagatctccaaggattcattttgacccatgacctcctccttctacagcccaactca
 P  P  D  L  Q  G  F  I  L  T  H  D  L  L  L  L  Q  P  N  S
atggaccctctccatgcctcctctcacacagcaaagatgaccacaaaacaagatgaactt
 M  D  P  L  H  A  S  S  H  T  A  K  M  T  T  K  Q  D  E  L
tacactgaattcttactaagcacacctcttgtgaagctaccagtgacttag
 Y  T  E  F  L  L  S  T  P  L  V  K  L  P  V  T  -
```

>PAX7_isoformWT2

atggctgcgctgcccgggaccgtcccgaggatgatgcgcccggcgcccggccagaactac
M A A L P G T V P R M M R P A P G Q N Y
ccgcgcaccggattccctctcgaagtctccacgcccctgggccaaggccgggtcaaccag
P R T G F P L E V S T P L G Q G R V N Q
ctcgggggggtcttcatcaacgggcggcccctgcccaaccacatccgccacaagatcgtg
L G G V F I N G R P L P N H I R H K I V
gagatggcccaccacggcatccggccctgcgtcatctcccggcagctgcgcgtctcccac
E M A H H G I R P C V I S R Q L R V S H
gggtgcgtctccaagatcctttgccgctaccaggagacgggctcgatccgccccggggcc
G C V S K I L C R Y Q E T G S I R P G A
atcggaggcagcaagcccaggcaggtcgcgactcccgacgtggagaaaaaaattgaagaa
I G G S K P R Q V A T P D V E K K I E E
tataagcgcgaaaatccaggcatgttcagctgggagatccgggaccggctcctcaaagac
Y K R E N P G M F S W E I R D R L L K D
ggccactgcgaccggagtacagtcccttcagtgagttcaatcagccgtgtgctccgaatc
G H C D R S T V P S V S S I S R V L R I
aagttcgggaagaaggaggaggaggaagattgcgacaagaaagaggaggatggcgagaag
K F G K K E E E E D C D K K E E D G E K
aaagccaagcacagcatcgacggcatcctgggagataaaggtaaccgactggatgaaggc
K A K H S I D G I L G D K G N R L D E G
tctgacgtcgagtctgaaccggacctgcccctgaagcgcaagcagcgccgcagccggacc
S D V E S E P D L P L K R K Q R R S R T
accttcactgcggaacagttggaggagctggagaaggcctttgagaggacccattacccg
T F T A E Q L E E L E K A F E R T H Y P
gatatctacacccgagaagagcttgctcagcggaccaaactcactgaggcccgggtccag
D I Y T R E E L A Q R T K L T E A R V Q
gtctggttcagcaaccgaagagcacggtggcgtaagcaagcaggagccaaccagctagca
V W F S N R R A R W R K Q A G A N Q L A
gcttttaaccacctcttgccaggggggcttcccacccactgggatgccagctttgccaccc
A F N H L L P G G F P P T G M P A L P P
tatcagcttccagattcgacctacccaacagccgccatttcccaagacggggggcagcacc
Y Q L P D S T Y P T A A I S Q D G G S T
gtgcacagaccccagcctctcccgccgtccaccatgcaccaggggggattggcagccgcc
V H R P Q P L P P S T M H Q G G L A A A
gccgccgactccagctcagcgtatggggcccggcacagcttttccagctactcggacacc
A A D S S S A Y G A R H S F S S Y S D T
ttcatgaaccctgcagctcccacaaaccacatgaaccccgtcagcaacggcctctcccct
F M N P A A P T N H M N P V S N G L S P
caggtgatgagcatcctcagcaaccccagtggagtcccaccgcagccccagggcgacttt
Q V M S I L S N P S G V P P Q P Q G D F
tccatctccccccctccacggcagcttggacactgccaattccatctcggccagctgcagc
S I S P L H G S L D T A N S I S A S C S
cagaggagcgactccatcaagtccgtcgacagcctccccacttcacaatcctactgtccc
Q R S D S I K S V D S L P T S Q S Y C P
ccgacctacaccaccactggctacagcgtggaccccgtggcaggctaccagtatgggcaa
P T Y T T T G Y S V D P V A G Y Q Y G Q
tatgggcaaactgcagttgactacctggccaaaaacgtgagcctgtctacacaacgcaga
Y G Q T A V D Y L A K N V S L S T Q R R
atgaagctcggggagcattccgctgtgttaggcctcctacccgtggaaacgggccaagct
M K L G E H S A V L G L L P V E T G Q A
tac**taa**tggagacacttgcagtctgctatcaaggagaagaaaaatccccctctgccacca
Y **-** W R H L Q S A I K E K K N P P L P P
gatctccaaggattcattttgacccatgacctcctccttctacagcccaactcaatggac
D L Q G F I L T H D L L L L Q P N S M D
cctctccatgcctcctctcacacagcaaagatgaccacaaaacaagatgaactttacact
P L H A S S H T A K M T T K Q D E L Y T
gaattcttactaagcacacctcttgtgaagctaccagtgacttag
E F L L S T P L V K L P V T -

>PAX7_isoformWT3

```
atggctgcgctgcccgggaccgtcccgaggatgatgcgcccggcgcccggccagaactac
 M  A  A  L  P  G  T  V  P  R  M  M  R  P  A  P  G  Q  N  Y 
ccgcgcaccggattccctctcgaagtctccacgcccctgggccaaggccgggtcaaccag
 P  R  T  G  F  P  L  E  V  S  T  P  L  G  Q  G  R  V  N  Q 
ctcgggggggtcttcatcaacgggcggcccctgcccaaccacatccgccacaagatcgtg
 L  G  G  V  F  I  N  G  R  P  L  P  N  H  I  R  H  K  I  V 
gagatggcccaccacggcatccggccctgcgtcatctcccggcagctgcgcgtctcccac
 E  M  A  H  H  G  I  R  P  C  V  I  S  R  Q  L  R  V  S  H 
gggtgcgtctccaagatcctttgccgctaccaggagacgggctcgatccgccccggggcc
 G  C  V  S  K  I  L  C  R  Y  Q  E  T  G  S  I  R  P  G  A 
atcggaggcagcaagcccagggtcgcgactcccgacgtggagaaaaaaattgaagaatat
 I  G  G  S  K  P  R  V  A  T  P  D  V  E  K  K  I  E  E  Y 
aagcgcgaaaatccaggcatgttcagctgggagatccgggaccggctcctcaaagacggc
 K  R  E  N  P  G  M  F  S  W  E  I  R  D  R  L  L  K  D  G 
cactgcgaccggagtacagtcccttcaggtttagtgagttcaatcagccgtgtgctccga
 H  C  D  R  S  T  V  P  S  G  L  V  S  S  I  S  R  V  L  R 
atcaagttcgggaagaaggaggaggaggaagattgcgacaagaaagaggaggatggcgag
 I  K  F  G  K  K  E  E  E  E  D  C  D  K  K  E  E  D  G  E 
aagaaagccaagcacagcatcgacggcatcctgggagataaaggtaaccgactggatgaa
 K  K  A  K  H  S  I  D  G  I  L  G  D  K  G  N  R  L  D  E 
ggctctgacgtcgagtctgaaccggacctgcccctgaagcgcaagcagcgccgcagccgg
 G  S  D  V  E  S  E  P  D  L  P  L  K  R  K  Q  R  R  S  R 
accaccttcactgcggaacagttggaggagctggagaaggcctttgagaggacccattac
 T  T  F  T  A  E  Q  L  E  E  L  E  K  A  F  E  R  T  H  Y 
ccggatatctacacccgagaagagcttgctcagcggaccaaactcactgaggcccgggtc
 P  D  I  Y  T  R  E  E  L  A  Q  R  T  K  L  T  E  A  R  V 
caggtctggttcagcaaccgaagagcacggtggcgtaagcaagcaggagccaaccagcta
 Q  V  W  F  S  N  R  R  A  R  W  R  K  Q  A  G  A  N  Q  L 
gcagcttttaaccacctcttgccagggggcttcccacccactgggatgccagctttgcca
 A  A  F  N  H  L  L  P  G  G  F  P  P  T  G  M  P  A  L  P 
ccctatcagcttccagattcgacctacccaacagccgccatttcccaagacgggggcagc
 P  Y  Q  L  P  D  S  T  Y  P  T  A  A  I  S  Q  D  G  G  S 
accgtgcacagaccccagcctctcccgccgtccaccatgcaccagggggggattggcagcc
 T  V  H  R  P  Q  P  L  P  P  S  T  M  H  Q  G  G  L  A  A 
gccgccgccgactccagctcagcgtatgggcccggcacagcttttccagctactcggac
 A  A  A  D  S  S  S  A  Y  G  A  R  H  S  F  S  S  Y  S  D 
accttcatgaaccctgcagctcccacaaaccacatgaaccccgtcagcaacggcctctcc
 T  F  M  N  P  A  A  P  T  N  H  M  N  P  V  S  N  G  L  S 
cctcaggtgatgagcatcctcagcaaccccagtggagtcccaccgcagccccagggcgac
 P  Q  V  M  S  I  L  S  N  P  S  G  V  P  P  Q  P  Q  G  D 
ttttccatctccccctccacggcagcttggacactgccaattccatctcggccagctgc
 F  S  I  S  P  L  H  G  S  L  D  T  A  N  S  I  S  A  S  C 
agccagaggagcgactccatcaagtccgtcgacagcctccccacttcacaatcctactgt
 S  Q  R  S  D  S  I  K  S  V  D  S  L  P  T  S  Q  S  Y  C 
cccccgacctacaccaccactggctacagcgtggaccccgtggcaggctaccagtatggg
 P  P  T  Y  T  T  T  G  Y  S  V  D  P  V  A  G  Y  Q  Y  G 
caatatgggcaaactgcagttgactacctggccaaaaacgtgagcctgtctacacaacgc
 Q  Y  G  Q  T  A  V  D  Y  L  A  K  N  V  S  L  S  T  Q  R 
agaatgaagctcggggagcattccgctgtgttaggcctcctacccgtggaaacgggccaa
 R  M  K  L  G  E  H  S  A  V  L  G  L  L  P  V  E  T  G  Q 
gcttactaatggagacacttgcagtctgctatcaaggagaagaaaaatccccctctgcca
 A  Y  -  W  R  H  L  Q  S  A  I  K  E  K  K  N  P  P  L  P 
ccagatctccaaggattcattttgacccatgacctcctccttctacagcccaactcaatg
 P  D  L  Q  G  F  I  L  T  H  D  L  L  L  L  Q  P  N  S  M 
gaccctctccatgcctcctctcacacagcaaagatgaccacaaaacaagatgaactttac
 D  P  L  H  A  S  S  H  T  A  K  M  T  T  K  Q  D  E  L  Y 
actgaattcttactaagcacacctcttgtgaagctaccagtgacttag
 T  E  F  L  L  S  T  P  L  V  K  L  P  V  T  - 
```

>PAX7_isoformWT4

```
atggctgcgctgcccgggaccgtcccgaggatgatgcgcccggcgcccggccagaactac
 M  A  A  L  P  G  T  V  P  R  M  M  R  P  A  P  G  Q  N  Y
ccgcgcaccggattccctctcgaagcaggtcgcgactcccgacgtggagaaaaaaattga
 P  R  T  G  F  P  L  E  A  G  R  D  S  R  R  G  E  K  N  -
agaatataagcgcgaaaatccaggcatgttcagctgggagatccgggaccggctcctcaa
 R  I  -  A  R  K  S  R  H  V  Q  L  G  D  P  G  P  A  P  Q
agacggccactgcgaccggagtacagtcccttcaggtttagtgagttcaatcagccgtgt
 R  R  P  L  R  P  E  Y  S  P  F  R  F  S  E  F  N  Q  P  C
gctccgaatcaagttcgggaagaaggaggaggaggaagattgcgacaagaaagaggagga
 A  P  N  Q  V  R  E  E  G  G  G  G  R  L  R  Q  E  R  G  G
tggcgagaagaaagccaagcacagcatcgacggcatcctgggagataaaggtaaccgact
 W  R  E  E  S  Q  A  Q  H  R  R  H  P  G  R  -  R  -  P  T
ggatgaaggctctgacgtcgagtctgaaccggacctgcccctgaagcgcaagcagcgccg
 G  -  R  L  -  R  R  V  -  T  G  P  A  P  E  A  Q  A  A  P
cagccggaccaccttcactgcggaacagttggaggagctggagaaggcctttgagaggac
 Q  P  D  H  L  H  C  G  T  V  G  G  A  G  E  G  L  -  E  D
ccattacccggatatctacacccgagaagagcttgctcagcggaccaaactcactgaggc
 P  L  P  G  Y  L  H  P  R  R  A  C  S  A  D  Q  T  H  -  G
ccgggtccaggtctggttcagcaaccgaagagcacggtggcgtaagcaagcaggagccaa
 P  G  P  G  L  V  Q  Q  P  K  S  T  V  A  -  A  S  R  S  Q
ccagctagcagcttttaaccacctcttgccaggggcttcccacccactgggatgccagc
 P  A  S  S  F  -  P  P  L  A  R  G  L  P  T  H  W  D  A  S
tttgccaccctatcagcttccagattcgacctacccaacagccgccatttcccaagacgg
 F  A  T  L  S  A  S  R  F  D  L  P  N  S  R  H  F  P  R  R
gggcagcaccgtgcacagaccccagcctctcccgccgtccaccatgcaccagggggggatt
 G  Q  H  R  A  Q  T  P  A  S  P  A  V  H  H  A  P  G  G  I
ggcagccgccgccgccgactccagctcagcgtatggggcccggcacagcttttccagcta
 G  S  R  R  R  R  L  Q  L  S  V  W  G  P  A  Q  L  F  Q  L
ctcggacaccttcatgaaccctgcagctcccacaaaccacatgaaccccgtcagcaacgg
 L  G  H  L  H  E  P  C  S  S  H  K  P  H  E  P  R  Q  Q  R
cctctcccctcaggtgatgagcatcctcagcaaccccagtggagtcccaccgcagcccca
 P  L  P  S  G  D  E  H  P  Q  Q  P  Q  W  S  P  T  A  A  P
gggcgacttttccatctccccccctccacggcagcttggacactgccaattccatctcggc
 G  R  L  F  H  L  P  P  P  R  Q  L  G  H  C  Q  F  H  L  G
cagctgcagccagaggagcgactccatcaagtccgtcgacagcctccccacttcacaatc
 Q  L  Q  P  E  E  R  L  H  Q  V  R  R  Q  P  P  H  F  T  I
ctactgtcccccgacctacaccaccactggctacagcgtggaccccgtggcaggctacca
 L  L  S  P  D  L  H  H  H  W  L  Q  R  G  P  R  G  R  L  P
gtatgggcaatatgggcaaactgcagttgactacctggccaaaaacgtgagcctgtctac
 V  W  A  I  W  A  N  C  S  -  L  P  G  Q  K  R  E  P  V  Y
acaacgcagaatgaagctcggggagcattccgctgtgttaggcctcctacccgtggaaac
 T  T  Q  N  E  A  R  G  A  F  R  C  V  R  P  P  T  R  G  N
gggccaagcttactaatggagacacttgcagtctgctatcaaggagaagaaaaatccccc
 G  P  S  L  L  M  E  T  L  A  V  C  Y  Q  G  E  E  K  S  P
tctgccaccagatctccaaggattcattttgacccatgacctcctccttctacagcccaa
 S  A  T  R  S  P  R  I  H  F  D  P  -  P  P  P  S  T  A  Q
ctcaatggaccctctccatgcctcctctcacacagcaaagatgaccacaaaacaagatga
 L  N  G  P  S  P  C  L  L  S  H  S  K  D  D  H  K  T  R  -
actttacactgaattcttactaagcacacctcttgtgaagctaccagtgacttag
 T  L  H  -  I  L  T  K  H  T  S  C  E  A  T  S  D  L
```

Sequence of the leopard gecko Mack Super Snow *PAX7* isoforms. The protein translation is provided. The starting site is highlighted in green, while the stop codon is in red.

>PAX7_isoformMSS1

```
atggctgcgctgcccgggaccgtcccgaggatgatgcgcccggcgcccggccagaactac
 M  A  A  L  P  G  T  V  P  R  M  M  R  P  A  P  G  Q  N  Y
ccgcgcaccggattccctctcgaagtctccacgcccctgggccaaggccgggtcaaccag
 P  R  T  G  F  P  L  E  V  S  T  P  L  G  Q  G  R  V  N  Q
ctcgggggggtcttcatcaacgggcggcccctgcccaaccacatccgccacaagatcgtg
 L  G  G  V  F  I  N  G  R  P  L  P  N  H  I  R  H  K  I  V
gagatggcccaccacggcatccggccctgcgtcatctcccggcagctgcgcgtctcccac
 E  M  A  H  H  G  I  R  P  C  V  I  S  R  Q  L  R  V  S  H
gggtgcgtctccaagatcctttgccgctaccaggagacgggctcgatccgccccggggcc
 G  C  V  S  K  I  L  C  R  Y  Q  E  T  G  S  I  R  P  G  A
atcggaggcagcaagcccaggcaggtcgcgactcccgacgtggagaaaaaaattgaagaa
 I  G  G  S  K  P  R  Q  V  A  T  P  D  V  E  K  K  I  E  E
tataagcgcgaaaatccaggcatgttcagctgggagatccgggaccggctcctcaaagac
 Y  K  R  E  N  P  G  M  F  S  W  E  I  R  D  R  L  L  K  D
ggccactgcgaccggagtacagtcccttcaggtaaccgcctggacgaaggctctgacgtc
 G  H  C  D  R  S  T  V  P  S  G  N  R  L  D  E  G  S  D  V
gagtctgaaccggacctgcccctgaagcgcaagcagcgccgcagccggaccaccttcact
 E  S  E  P  D  L  P  L  K  R  K  Q  R  R  S  R  T  T  F  T
gcggaacagttggaggagctggagaaggcctttgagaggacccattacccggatatctac
 A  E  Q  L  E  E  L  E  K  A  F  E  R  T  H  Y  P  D  I  Y
acccgagaagagcttgcccagcggaccaagctcactgaggcccgggtccaggtctggttc
 T  R  E  E  L  A  Q  R  T  K  L  T  E  A  R  V  Q  V  W  F
agcaaccgaagagcacggtggcgtaagcaagcaggagccaaccagctagcagcttttaac
 S  N  R  R  A  R  W  R  K  Q  A  G  A  N  Q  L  A  A  F  N
cacctcttgccaggggggcttcccacccactgggatgccagctttgccaccctatcagctt
 H  L  L  P  G  G  F  P  P  T  G  M  P  A  L  P  P  Y  Q  L
ccagattcgacctacccaacagccgccatttcccaagacgggggcagcaccgtgcacaga
 P  D  S  T  Y  P  T  A  A  I  S  Q  D  G  G  S  T  V  H  R
ccccagcctctcccgccgtccaccatgcaccaggggggattggcagccgccgccgccgac
 P  Q  P  L  P  P  S  T  M  H  Q  G  G  L  A  A  A  A  A  D
tccagctcagcgtatggggcccggcacagcttttccagctactcggacaccttcatgaac
 S  S  S  A  Y  G  A  R  H  S  F  S  S  Y  S  D  T  F  M  N
cctgcagctcccacaaaccacatgaaccccgtcagcaacggcctctcccctcaggtgatg
 P  A  A  P  T  N  H  M  N  P  V  S  N  G  L  S  P  Q  V  M
agcatcctcagcaaccccagtggagtcccaccgcagccccagggcgacttttccatctcc
 S  I  L  S  N  P  S  G  V  P  P  Q  P  Q  G  D  F  S  I  S
cccctccacggcagcttggacactgccaattccatctcggccagctgcagccagaggagc
 P  L  H  G  S  L  D  T  A  N  S  I  S  A  S  C  S  Q  R  S
gactccatcaagtccgtcgacagcctccccacttcacaatcctactgtcccccgacctac
 D  S  I  K  S  V  D  S  L  P  T  S  Q  S  Y  C  P  P  T  Y
accaccactggctacagcgtggaccccgtggcaggctaccagtatgggcaatatgggcaa
 T  T  T  G  Y  S  V  D  P  V  A  G  Y  Q  Y  G  Q  Y  G  Q
actgcagttgactacctggccaaaaacgtgagcctgtctacacaacgcagaatgaagctc
 T  A  V  D  Y  L  A  K  N  V  S  L  S  T  Q  R  R  M  K  L
ggggagcattccgctgtgttaggcctcctacccgtggaaacgggccaagcttactaatgg
 G  E  H  S  A  V  L  G  L  L  P  V  E  T  G  Q  A  Y  -  W
agacacttgcagtctgctatcaaggagaagaaaaatcccccctctgccaccagatctccaa
 R  H  L  Q  S  A  I  K  E  K  K  N  P  P  L  P  P  D  L  Q
ggattcattttgacccatgacctcctccttctacagcccaactcaatggaccctctccat
 G  F  I  L  T  H  D  L  L  L  L  Q  P  N  S  M  D  P  L  H
gcctcctctcacacagcaaagatgaccacaaaacaagatgaactttacactgaattctta
 A  S  S  H  T  A  K  M  T  T  K  Q  D  E  L  Y  T  E  F  L
ctaagcacacctcttgtgaagctaccagtgacttag
 L  S  T  P  L  V  K  L  P  V  T  -
```

```
>PAX7_isoformMSS2
atggctgcgctgcccgggaccgtcccgaggatgatgcgcccggcgcccggccagaactac
 M  A  A  L  P  G  T  V  P  R  M  M  R  P  A  P  G  Q  N  Y
ccgcgcaccggattccctctcgaagcaggtcgcgactcccgacgtggagaaaaaaattga
 P  R  T  G  F  P  L  E  A  G  R  D  S  R  R  G  E  K  N  -
agaatataagcgcgaaaatccaggcatgttcagctgggagatccgggaccggctcctcaa
 R  I  -  A  R  K  S  R  H  V  Q  L  G  D  P  G  P  A  P  Q
agacggccactgcgaccggagtacagtcccttcaggtaaccgcctggacgaaggctctga
 R  R  P  L  R  P  E  Y  S  P  F  R  -  P  P  G  R  R  L  -
cgtcgagtctgaaccggacctgcccctgaagcgcaagcagcgccgcagccggaccacctt
 R  R  V  -  T  G  P  A  P  E  A  Q  A  A  P  Q  P  D  H  L
cactgcggaacagttggaggagctggagaaggcctttgagaggacccattacccggatat
 H  C  G  T  V  G  G  A  G  E  G  L  -  E  D  P  L  P  G  Y
ctacacccgagaagagcttgcccagcggaccaagctcactgaggcccgggtccaggtctg
 L  H  P  R  R  A  C  P  A  D  Q  A  H  -  G  P  G  P  G  L
gttcagcaaccgaagagcacggtggcgtaagcaagcaggagccaaccagctagcagcttt
 V  Q  Q  P  K  S  T  V  A  -  A  S  R  S  Q  P  A  S  S  F
taaccacctcttgccaggggggcttcccacccactgggatgccagctttgccaccctatca
 -  P  P  L  A  R  G  L  P  T  H  W  D  A  S  F  A  T  L  S
gcttccagattcgacctacccaacagccgccatttcccaagacgggggcagcaccgtgca
 A  S  R  F  D  L  P  N  S  R  H  F  P  R  R  G  Q  H  R  A
cagaccccagcctctcccgccgtccaccatgcaccagggggggattggcagccgccgccgc
 Q  T  P  A  S  P  A  V  H  H  A  P  G  G  I  G  S  R  R  R
cgactccagctcagcgtatgggcccggcacagcttttccagctactcggacaccttcat
 R  L  Q  L  S  V  W  G  P  A  Q  L  F  Q  L  L  G  H  L  H
gaaccctgcagctcccacaaaccacatgaaccccgtcagcaacggcctctcccctcaggt
 E  P  C  S  S  H  K  P  H  E  P  R  Q  Q  R  P  L  P  S  G
gatgagcatcctcagcaaccccagtggagtcccaccgcagccccagggcgacttttccat
 D  E  H  P  Q  Q  P  Q  W  S  P  T  A  A  P  G  R  L  F  H
ctccccccctccacggcagcttggacactgccaattccatctcggccagctgcagccagag
 L  P  P  P  R  Q  L  G  H  C  Q  F  H  L  G  Q  L  Q  P  E
gagcgactccatcaagtccgtcgacagcctccccacttcacaatcctactgtcccccgac
 E  R  L  H  Q  V  R  R  Q  P  P  H  F  T  I  L  L  S  P  D
ctacaccaccactggctacagcgtggaccccgtggcaggctaccagtatgggcaatatgg
 L  H  H  H  W  L  Q  R  G  P  R  G  R  L  P  V  W  A  I  W
gcaaactgcagttgactacctggccaaaaacgtgagcctgtctacacaacgcagaatgaa
 A  N  C  S  -  L  P  G  Q  K  R  E  P  V  Y  T  T  Q  N  E
gctcggggagcattccgctgtgttaggcctcctacccgtggaaacgggccaagcttacta
 A  R  G  A  F  R  C  V  R  P  P  T  R  G  N  G  P  S  L  L
atggagacacttgcagtctgctatcaaggagaagaaaaatccccctctgccaccagatct
 M  E  T  L  A  V  C  Y  Q  G  E  E  K  S  P  S  A  T  R  S
ccaaggattcattttgacccatgacctcctccttctacagcccaactcaatggaccctct
 P  R  I  H  F  D  P  -  P  P  P  S  T  A  Q  L  N  G  P  S
ccatgcctcctctcacacagcaaagatgaccacaaaacaagatgaactttacactgaatt
 P  C  L  L  S  H  S  K  D  D  H  K  T  R  -  T  L  H  -  I
cttactaagcacacctcttgtgaagctaccagtgacttag
 L  T  K  H  T  S  C  E  A  T  S  D  L
```

```
>PAX7_isoformMSS3
atggctgcgctgcccgggaccgtcccgaggatgatgcgcccggcgcccggccagaactac
 M  A  A  L  P  G  T  V  P  R  M  M  R  P  A  P  G  Q  N  Y
ccgcgcaccggattccctctcgaagtctccacgcccctgggccaaggccgggtcaaccag
 P  R  T  G  F  P  L  E  V  S  T  P  L  G  Q  G  R  V  N  Q
ctcgggggggtcttcatcaacgggcggcccctgcccaaccacatccgccacaagatcgtg
 L  G  G  V  F  I  N  G  R  P  L  P  N  H  I  R  H  K  I  V
gagatggcccaccacggcatccggccctgcgtcatctcccggcagctgcgcgtctcccac
 E  M  A  H  H  G  I  R  P  C  V  I  S  R  Q  L  R  V  S  H
gggtgcgtctccaagatcctttgccgctaccaggagacgggctcgatccgcctcggggcc
 G  C  V  S  K  I  L  C  R  Y  Q  E  T  G  S  I  R  L  G  A
atcggaggcagcaagcccagggtcgcgactcccgacgtggagaaaaaaattgaagaatat
 I  G  G  S  K  P  R  V  A  T  P  D  V  E  K  K  I  E  E  Y
aagcgcgaaaatccaggcatgttcagctgggagatccgggaccggctcctcaaagacggc
 K  R  E  N  P  G  M  F  S  W  E  I  R  D  R  L  L  K  D  G
cactgcgaccggagtacagtcccttcaggtaaccgcctggacgaaggctctgacgtcgag
 H  C  D  R  S  T  V  P  S  G  N  R  L  D  E  G  S  D  V  E
tctgaaccggacctgcccctgaagcgcaagcagcgccgcagccggaccaccttcactgcg
 S  E  P  D  L  P  L  K  R  K  Q  R  R  S  R  T  T  F  T  A
gaacagttggaggagctggagaaggcctttgagaggacccattacccggatatctacacc
 E  Q  L  E  E  L  E  K  A  F  E  R  T  H  Y  P  D  I  Y  T
cgagaagagcttgcccagcggaccaagctcactgaggcccgggtccaggtctggttcagc
 R  E  E  L  A  Q  R  T  K  L  T  E  A  R  V  Q  V  W  F  S
aaccgaagagcacggtggcgtaagcaagcaggagccaaccagctagcagcttttaaccac
 N  R  R  A  R  W  R  K  Q  A  G  A  N  Q  L  A  A  F  N  H
ctcttgccaggggggcttcccacccactgggatgccagctttgccaccctatcagcttcca
 L  L  P  G  G  F  P  P  T  G  M  P  A  L  P  P  Y  Q  L  P
gattcgacctacccaacagccgccatttcccaagacgggggcagcaccgtgcacagaccc
 D  S  T  Y  P  T  A  A  I  S  Q  D  G  G  S  T  V  H  R  P
cagcctctcccgccgtccaccatgcaccagggggggattggcagccgccgccgccgactcc
 Q  P  L  P  P  S  T  M  H  Q  G  G  L  A  A  A  A  A  D  S
agctcagcgtatggggcccggcacagcttttccagctactcggacaccttcatgaaccct
 S  S  A  Y  G  A  R  H  S  F  S  S  Y  S  D  T  F  M  N  P
gcagctcccacaaaccacatgaaccccgtcagcaacggcctctcccctcaggtgatgagc
 A  A  P  T  N  H  M  N  P  V  S  N  G  L  S  P  Q  V  M  S
atcctcagcaaccccagtggagtcccaccgcagccccagggcgacttttccatctccccc
 I  L  S  N  P  S  G  V  P  P  Q  P  Q  G  D  F  S  I  S  P
ctccacggcagcttggacactgccaattccatctcggccagctgcagccagaggagcgac
 L  H  G  S  L  D  T  A  N  S  I  S  A  S  C  S  Q  R  S  D
tccatcaagtccgtcgacagcctccccacttcacaatcctactgtcccccgacctacacc
 S  I  K  S  V  D  S  L  P  T  S  Q  S  Y  C  P  P  T  Y  T
accactggctacagcgtggaccccgtggcaggctaccagtatgggcaatatgggcaaact
 T  T  G  Y  S  V  D  P  V  A  G  Y  Q  Y  G  Q  Y  G  Q  T
gcagttgactacctggccaaaaacgtgagcctgtctacacaacgcagaatgaagctcggg
 A  V  D  Y  L  A  K  N  V  S  L  S  T  Q  R  R  M  K  L  G
gagcattccgctgtgttaggcctcctacccgtggaaacgggccaagcttactaatggaga
 E  H  S  A  V  L  G  L  L  P  V  E  T  G  Q  A  Y  -  W  R
cacttgcagtctgctatcaaggagaagaaaaatcccccctctgccaccagatctccaagga
 H  L  Q  S  A  I  K  E  K  K  N  P  P  L  P  P  D  L  Q  G
ttcattttgacccatgacctcctccttctacagcccaactcaatggaccctctccatgcc
 F  I  L  T  H  D  L  L  L  L  Q  P  N  S  M  D  P  L  H  A
tcctctcacacagcaaagatgaccacaaaacaagatgaactttacactgaattcttacta
 S  S  H  T  A  K  M  T  T  K  Q  D  E  L  Y  T  E  F  L  L
agcacacctcttgtgaagctaccagtgacttag
 S  T  P  L  V  K  L  P  V  T  -
```

>PAX7_isoformMSS4

```
atggctgcgctgcccgggaccgtcccgaggatgatgcgcccggcgcccggccagaactac
 M  A  A  L  P  G  T  V  P  R  M  M  R  P  A  P  G  Q  N  Y
ccgcgcaccggattccctctcgaagtctccacgcccctgggccaaggccgggtcaaccag
 P  R  T  G  F  P  L  E  V  S  T  P  L  G  Q  G  R  V  N  Q
ctcggggggtcttcatcaacgggcggcccctgcccaaccacatccgccacaagatcgtg
 L  G  G  V  F  I  N  G  R  P  L  P  N  H  I  R  H  K  I  V
gagatggcccaccacggcatccggccctgcgtcatctcccggcagctgcgcgtctcccac
 E  M  A  H  H  G  I  R  P  C  V  I  S  R  Q  L  R  V  S  H
gggtgcgtctccaagatcctttgccgctaccaggagacgggctcgatccgccccggggcc
 G  C  V  S  K  I  L  C  R  Y  Q  E  T  G  S  I  R  P  G  A
atcggaggcagcaagcccaggcaggtcgcgactcccgacgtggagaaaaaaattgaagaa
 I  G  G  S  K  P  R  Q  V  A  T  P  D  V  E  K  K  I  E  E
tataagcgcgaaaatccaggcatgttcagctgggagatccgggaccggctcctcaaagac
 Y  K  R  E  N  P  G  M  F  S  W  E  I  R  D  R  L  L  K  D
ggccactgcgaccggagtacagtcccttcaggtttagtgagttcaatcagccgtgtgctc
 G  H  C  D  R  S  T  V  P  S  G  L  V  S  S  I  S  R  V  L
cgaatcaagttcgggaagaaggaggaggaggaagattgcgacaagaaagaggaggatggc
 R  I  K  F  G  K  K  E  E  E  E  D  C  D  K  K  E  E  D  G
gagaagaaagccaagcacagcatcgacggcatcctgggagcccagcgaggctcccctctc
 E  K  K  A  K  H  S  I  D  G  I  L  G  A  Q  R  G  S  P  L
ctcctaaagaattggacccccctccccagcagacctacttcctcctaaactccgagggcgt
 L  L  K  N  W  T  P  S  P  A  D  L  L  P  P  K  L  R  G  R
acttctgcttggggagtcgctggtagtttccttctatggtaaccgcctggacgaaggctc
 T  S  A  W  G  V  A  G  S  F  L  L  W  -  P  P  G  R  R  L
tgacgtcgagtctgaaccggacctgcccctgaagcgcaagcagcgccgcagccggaccac
 -  R  R  V  -  T  G  P  A  P  E  A  Q  A  A  P  Q  P  D  H
cttcactgcggaacagttggaggagctggagaaggcctttgagaggacccattacccgga
 L  H  C  G  T  V  G  G  A  G  E  G  L  -  E  D  P  L  P  G
tatctacacccgagaagagcttgcccagcggaccaagctcactgaggcccgggtccaggt
 Y  L  H  P  R  R  A  C  P  A  D  Q  A  H  -  G  P  G  P  G
ctggttcagcaaccgaagagcacggtggcgtaagcaagcaggagccaaccagctagcagc
 L  V  Q  Q  P  K  S  T  V  A  -  A  S  R  S  Q  P  A  S  S
ttttaaccacctcttgccaggggcttcccacccactgggatgccagctttgccaccctа
 F  -  P  P  L  A  R  G  L  P  T  H  W  D  A  S  F  A  T  L
tcagcttccagattcgacctacccaacagccgccatttcccaagacgggggcagcaccgt
 S  A  S  R  F  D  L  P  N  S  R  H  F  P  R  R  G  Q  H  R
gcacagaccccagcctctcccgccgtccaccatgcaccagggggattggcagccgccgc
 A  Q  T  P  A  S  P  A  V  H  H  A  P  G  G  I  G  S  R  R
cgccgactccagctcagcgtatggggcccggcacagcttttccagctactcggacacctt
 R  R  L  Q  L  S  V  W  G  P  A  Q  L  F  Q  L  L  G  H  L
catgaaccctgcagctcccacaaaccacatgaaccccgtcagcaacggcctctcccctca
 H  E  P  C  S  S  H  K  P  H  E  P  R  Q  Q  R  P  L  P  S
ggtgatgagcatcctcagcaaccccagtggagtcccaccgcagccccagggcgactttc
 G  D  E  H  P  Q  Q  P  Q  W  S  P  T  A  A  P  G  R  L  F
catctccccccctccacggcagcttggacactgccaattccatctcggccagctgcagcca
 H  L  P  P  P  R  Q  L  G  H  C  Q  F  H  L  G  Q  L  Q  P
gaggagcgactccatcaagtccgtcgacagcctccccacttcacaatcctactgtccccc
 E  E  R  L  H  Q  V  R  R  Q  P  P  H  F  T  I  L  L  S  P
gacctacaccaccactggctacagcgtggaccccgtggcaggctaccagtatgggcaata
 D  L  H  H  H  W  L  Q  R  G  P  R  G  R  L  P  V  W  A  I
tgggcaaactgcagttgactacctggccaaaaacgtgagcctgtctacacaacgcagaat
 W  A  N  C  S  -  L  P  G  Q  K  R  E  P  V  Y  T  T  Q  N
gaagctcggggagcattccgctgtgttaggcctcctacccgtggaaacgggccaagctta
 E  A  R  G  A  F  R  C  V  R  P  P  T  R  G  N  G  P  S  L
ctaatggagacacttgcagtctgctatcaaggagaagaaaaatccccctctgccaccaga
 L  M  E  T  L  A  V  C  Y  Q  G  E  E  K  S  P  S  A  T  R
tctccaaggattcattttgacccatgacctcctccttctacagcccaactcaatggaccc
 S  P  R  I  H  F  D  P  -  P  P  P  S  T  A  Q  L  N  G  P
tctccatgcctcctctcacacagcaaagatgaccacaaaacaagatgaactttacactga
 S  P  C  L  L  S  H  S  K  D  D  H  K  T  R  -  T  L  H  -
attcttactaagcacacctcttgtgaagctaccagtgacttag
 I  L  T  K  H  T  S  C  E  A  T  S  D  L
```

>PAX7_isoformMSS5

```
atggctgcgctgcccgggaccgtcccgaggatgatgcgcccggcgcccggccagaactac
 M  A  A  L  P  G  T  V  P  R  M  M  R  P  A  P  G  Q  N  Y 
ccgcgcaccggattccctctcgaagtctccacgcccctgggccaaggccgggtcaaccag
 P  R  T  G  F  P  L  E  V  S  T  P  L  G  Q  G  R  V  N  Q 
ctcggggggtcttcatcaacgggcggcccctgcccaaccacatccgccacaagatcgtg
 L  G  G  V  F  I  N  G  R  P  L  P  N  H  I  R  H  K  I  V 
gagatggcccaccacggcatccggccctgcgtcatctcccggcagctgcgcgtctcccac
 E  M  A  H  H  G  I  R  P  C  V  I  S  R  Q  L  R  V  S  H 
gggtgcgtctccaagatcctttgccgctaccaggagacgggctcgatccgccccggggcc
 G  C  V  S  K  I  L  C  R  Y  Q  E  T  G  S  I  R  P  G  A 
atcggaggcagcaagcccaggcaggtcgcgactcccgacgtggagaaaaaaattgaagaa
 I  G  G  S  K  P  R  Q  V  A  T  P  D  V  E  K  K  I  E  E 
tataagcgcgaaaatccaggcatgttcagctgggagatccgggaccggctcctcaaagac
 Y  K  R  E  N  P  G  M  F  S  W  E  I  R  D  R  L  L  K  D 
ggccactgcgaccggagtacagtcccttcagtgagttcaatcagccgtgtgctccgaatc
 G  H  C  D  R  S  T  V  P  S  V  S  S  I  S  R  V  L  R  I 
aagttcgggaagaaggaggaggaggaagattgcgacaagaaagaggaggatggcgagaag
 K  F  G  K  K  E  E  E  E  D  C  D  K  K  E  E  D  G  E  K 
aaagccaagcacagcatcgacggcatcctgggagcccagcgaggctcccctctcctccta
 K  A  K  H  S  I  D  G  I  L  G  A  Q  R  G  S  P  L  L  L 
aagaattggacccccctccccagcagacctacttcctcctaaactccgagggcgtacttct
 K  N  W  T  P  S  P  A  D  L  L  P  P  K  L  R  G  R  T  S 
gcttggggagtcgctggtagtttccttctatggtaaccgcctggacgaaggctctgacgt
 A  W  G  V  A  G  S  F  L  L  W  -  P  P  G  R  R  L  -  R 
cgagtctgaaccggacctgcccctgaagcgcaagcagcgccgcagccggaccaccttcac
 R  V  -  T  G  P  A  P  E  A  Q  A  A  P  Q  P  D  H  L  H 
tgcggaacagttggaggagctggagaaggcctttgagaggacccattacccggatatcta
 C  G  T  V  G  G  A  G  E  G  L  -  E  D  P  L  P  G  Y  L 
cacccgagaagagcttgcccagcggaccaagctcactgaggcccgggtccaggtctggtt
 H  P  R  R  A  C  P  A  D  Q  A  H  -  G  P  G  P  G  L  V 
cagcaaccgaagagcacggtggcgtaagcaagcaggagccaaccagctagcagcttttaa
 Q  Q  P  K  S  T  V  A  -  A  S  R  S  Q  P  A  S  S  F  - 
ccacctcttgccaggggggcttcccacccactgggatgccagctttgccaccctatcagct
 P  P  L  A  R  G  L  P  T  H  W  D  A  S  F  A  T  L  S  A 
tccagattcgacctacccaacagccgccatttcccaagacgggggcagcaccgtgcacag
 S  R  F  D  L  P  N  S  R  H  F  P  R  R  G  Q  H  R  A  Q 
accccagcctctcccgccgtccaccatgcaccagggggggattggcagccgccgccgccga
 T  P  A  S  P  A  V  H  H  A  P  G  G  I  G  S  R  R  R  R 
ctccagctcagcgtatggggcccggcacagcttttccagctactcggacaccttcatgaa
 L  Q  L  S  V  W  G  P  A  Q  L  F  Q  L  L  G  H  L  H  E 
ccctgcagctcccacaaaccacatgaaccccgtcagcaacggcctctcccctcaggtgat
 P  C  S  S  H  K  P  H  E  P  R  Q  Q  R  P  L  P  S  G  D 
gagcatcctcagcaaccccagtggagtcccaccgcagccccagggcgacttttccatctc
 E  H  P  Q  Q  P  Q  W  S  P  T  A  A  P  G  R  L  F  H  L 
ccccctccacggcagcttggacactgccaattccatctcggccagctgcagccagaggag
 P  P  P  R  Q  L  G  H  C  Q  F  H  L  G  Q  L  Q  P  E  E 
cgactccatcaagtccgtcgacagcctccccacttcacaatcctactgtcccccgaccta
 R  L  H  Q  V  R  R  Q  P  P  H  F  T  I  L  L  S  P  D  L 
caccaccactggctacagcgtggaccccgtggcaggctaccagtatgggcaatatgggca
 H  H  H  W  L  Q  R  G  P  R  G  R  L  P  V  W  A  I  W  A 
aactgcagttgactacctggccaaaaacgtgagcctgtctacacaacgcagaatgaagct
 N  C  S  -  L  P  G  Q  K  R  E  P  V  Y  T  T  Q  N  E  A 
cggggagcattccgctgtgttaggcctcctacccgtggaaacgggccaagcttactaatg
 R  G  A  F  R  C  V  R  P  P  T  R  G  N  G  P  S  L  L  M 
gagacacttgcagtctgctatcaaggagaagaaaaatccccctctgccaccagatctcca
 E  T  L  A  V  C  Y  Q  G  E  E  K  S  P  S  A  T  R  S  P 
aggattcattttgacccatgacctcctccttctacagcccaactcaatggaccctctcca
 R  I  H  F  D  P  -  P  P  P  S  T  A  Q  L  N  G  P  S  P 
tgcctcctctcacacagcaaagatgaccacaaaacaagatgaactttacactgaattctt
 C  L  L  S  H  S  K  D  D  H  K  T  R  -  T  L  H  -  I  L 
actaagcacacctcttgtgaagctaccagtgacttag
 T  K  H  T  S  C  E  A  T  S  D  L 
```
